# Effects of pitch size on soccer players’ physiological, physical, technical, and tactical responses during small-sided games: a meta-analytical comparison

**DOI:** 10.5114/biolsport.2023.110748

**Published:** 2022-01-21

**Authors:** Filipe Manuel Clemente, Gibson Moreira Praça, Rodrigo Aquino, Daniel Castillo, Javier Raya-González, Markel Rico-González, José Afonso, Hugo Sarmento, Ana Filipa Silva, Rui Silva, Rodrigo Ramirez-Campillo

**Affiliations:** 1 Escola Superior Desporto e Lazer, Instituto Politécnico de Viana do Castelo, Rua Escola Industrial e Comercial de Nun’Álvares, 4900-347 Viana do Castelo, Portugal; 2 Instituto de Telecomunicações, Delegação da Covilhã, Lisboa 1049-001, Portugal; 3 Sports Department, Universidade Federal de Minas Gerais, Belo Horizonte, Brazil; 4 Department of Sports, Center of Physical Education and Sports, Federal University of Espírito Santo, Vitória, Espírito Santo, Brazil; 5 Faculty of Education, Universidad de Valladolid, 42004, Soria, Spain; 6 Faculty of Health Sciences, Universidad Isabel I, Burgos, Spain; 7 Department of Physical Education and Sport, University of the Basque Country, UPV-EHU, Lasarte 71, 01007 Vitoria-Gasteiz, Spain; 8 Centre for Research, Education, Innovation and Intervention in Sport, Faculty of Sport of the University of Porto, Porto, Portugal; 9 University of Coimbra, Research Unit for Sport and Physical Activity. Faculty of Sport Sciences and Physical Education, Coimbra, Portugal; 10 The Research Centre in Sports Sciences, Health Sciences and Human Development (CIDESD), Vila Real 5001-801, Portugal; 11 Department of Physical Activity Sciences. Universidad de Los Lagos. Santiago, Chile; 12 Exercise and Rehabilitation Sciences Laboratory, School of Physical Therapy, Faculty of Rehabilitation Sciences, Universidad Andres Bello, Santiago 7591538, Chile

**Keywords:** Football, Soccer, Athletic performance, Human physical conditioning, Motor learning, Motor skills

## Abstract

One of the most often-used task constraints in designing small-sided games (SSGs) is the manipulation of pitch size to promote increases or decreases in the relative area per player. Such adjustments cause changes in the acute responses during SSGs. This systematic review with meta-analysis aimed to compare the effects of smaller vs. larger pitch sizes on soccer players’ physiological, physical, technical, and tactical responses during SSGs. Comparisons between smaller and larger pitches were not considered based on a specific size, but also between using at least two dimensions in the same comparative study, aiming to understand differences between using smaller and larger (independently of the specific dimensions). The data sources utilized were PubMed, PsycINFO, Scielo, Scopus, SPORTDiscus, and Web of Science. The database search initially yielded 249 titles. From those, 41 articles were eligible for the systematic review and meta-analysis. Results revealed that, compared to smaller pitches, SSGs played on larger pitches induced greater values for heart rate (p < 0.001; ES = 0.50), rate of perceived exertion (p < 0.001; ES = 0.70), total distance (p < 0.001; ES = 1.95), high-speed running (p < 0.001; ES = 1.20), stretch index (p < 0.001; ES = 1.02) and surface area (p < 0.001; ES = 1.54). No significant differences were found between pitch size regarding the numbers of accelerations (p = 0.232; ES = 0.45), decelerations (p = 0.111; ES = 0.85), passes (p = 0.897; ES = 0.02), dribbles (p = 0.823; ES = -0.05), or positional centroid (p = 0.053; ES = 0.56). Larger pitch sizes can be implemented as a meaningful task constraint to increase the internal and external load experienced by soccer players during SSGs, as well as to increase the dispersion of players while acting together. These results were found independent of format and age group.

## INTRODUCTION

Soccer is classified as an intermittent exercise [1] in which the effort exerted depends on the dynamic of the game [2]. Considering the complexity of soccer, performance is multidimensional—fitness status [3], technical skill [4], and tactical knowledge and execution [5] are just a few examples of parts that act concurrently to explain the ultimate outcome. Naturally, the aforementioned multidimensional factors also explain the physiological, physical, technical, and tactical responses of players training and matches [6].

The use of drill-based games such as small-sided games (SSGs), also known as small-sided conditioned games, have become popular since they reflect the multidimensional stimulus provided by matches while allowing the coach to alter players’ specific responses by manipulating various task constraints [7–9]. SSGs can be thought of as adjusted versions of official games in which coaches adjust specific constraints (or conditions) to change the behaviors of the players [10]. Among the most common adjustments used by coaches is the pitch configuration, as implementing smaller vs. larger pitch sizes impacts players’ behaviors [11].

Changing the pitch size (while keeping the same format of play) causes variations in relative area per player (calculated as the area of the pitch divided by the number of outfield players involved in the game) [12]. This manipulation is one of the main concerns while using SSGs since different relative areas per player for the same format change the players’ responses [13, 14]. Decreasing or increasing the relative area per player can initiate changes in physiological responses, physical demands, technical execution, and tactical behavior (as well as collective dynamics) [15–17]. If the information is not systematized (e.g., using a meta-analysis), it is difficult to understand the true effects of changing the pitch size since different moderators may compromise the findings.

Most of the original studies testing the effects of different pitch sizes on players’ responses have focused on specific measures within the main outcomes of physiological, physical, technical, and tactical responses [11]. In the case of physiological responses, the most often-used measures are heart rate, rate of perceived exertion (RPE), and blood lactate concentrations [14]. In the case of physical demands, microelectromechanical devices (e.g., Global Navigation Satellite System, Inertial Measurement Units) are usually used to assess the total distance covered, distances covered at different speed thresholds, and the number of accelerations/decelerations performed by players [18]. For technical execution, observational analysis is usually conducted to identify the number and accuracy of passes, receptions, dribbles, and shots during SSGs [15, 19]. Finally, in the case of tactical behavior also observational analysis is used to identify the accuracy of attacking and defensive behaviors or using bidimensional data to analyze measures related to the team’s spread or dispersion in the pitch [20].

Since adjustments in pitch size for the same format of play (e.g., 4 vs. 4 played on a smaller pitch (50 m^2^ per player) vs. a larger pitch (100 m^2^ per player) induce changes in players’ responses, it may be determinant to identify the impact of those changes. This allows coaches to understand the consequences of their adjustments on players’ responses and identify the most appropriate pitch sizes for specific objectives. Although systematic reviews have been conducted on the topic of SSGs (particularly summarizing the evidence regarding the impact of pitch size manipulation on players’ responses [7–9, 21, 22], no meta-analysis has been performed to identify the effects of smaller vs. larger pitch sizes on players’ responses. A meta-analysis may provide consistent evidence about the magnitudes of changes occurring between smaller and larger pitch sizes. Therefore, the purpose of this systematic review with meta-analysis was to compare the effects of smaller vs. larger pith sizes on physiological, physical, technical, and tactical responses during small-sided soccer games.

## MATERIALS AND METHODS

This systematic review and meta-analysis followed the Cochrane Collaboration [23], PRISMA (Preferred Reporting Items for Systematic Reviews and Meta-analyses) guidelines [24] and guidelines for performing systematic review in sports sciences [25]. The PICOS approach (Population, Intervention, Comparator, Outcomes, Study design) was followed: (P) soccer players from any age-group, sex or skill, without injury, illness or other clinical condition; (I) smaller pitch sizes using any format of play (number of players involved) or other task condition; (C) larger pitch sizes using any format of play (number of players involved) or other task condition (keeping the same experimental conditions of smaller formats); (O) mean and standard deviation (SD) values in both pitch sizes for, at least, one of the following main outcomes: physiological responses, physical responses, technical actions and tactical behaviors; and (S) counterbalanced cross-over design. Important to highlight those comparisons between smaller and larger pitches were not considered based on a specific size, but also between using at least two dimensions in the same comparative study, aiming to understand differences between using smaller and larger (independently of the specific dimensions). The protocol was registered with the International Platform of Registered Systematic Review and Meta-Analysis Protocols with the number INPLASY202140016 and the DOI number 10.37766/inplasy2021.4.0016.

### Eligibility criteria

Inclusion and exclusion criteria for this systematic review and meta-analysis can be found in table 1.

**TABLE 1:** Inclusion and exclusion criteria

Item	Inclusion criteria	Exclusion criteria
Population	Soccer players from any age-group, sex or skill, without injury, illness or other clinical condition.	Other sports than soccer (e.g., futsal or football indoor, beach soccer, American football, Australian football, basketball, handball, volleyball, hockey).

Intervention	Smaller pitch sizes using any format of play (number of players involved) or other task condition. The following conditions were ensured: The same pitch size was repeated at least two times (two repetitions) for the same players; The smaller pith size was extracted from the lowest relative pitch area (i.e., in case of studies comparing ≥ three pitch sizes for the same format or condition, only the smallest pitch size was extracted); The same experimental conditions between smaller and larger pitch sizes were ensured (i.e., same teams, same players, same time duration, same task constraints).	The same pitch size was applied in only one repetition; Smaller and larger pitch sizes conditions were not applied with same contextual and experimental conditions.

Comparator	Larger pitch sizes using any format of play (number of players involved) or other task condition. The following conditions were ensured: The same pitch size was repeated at least two times (two repetitions) for the same players; The larger pith size was extracted from the greatest relative pitch area (i.e., in case of studies comparing ≥ three pitch sizes for the same format or condition, only the largest pitch size was extracted); The same experimental conditions between smaller and larger pitch sizes were ensured (i.e., same teams, same players, same time duration, same task constraints).	The same pitch size was applied in only one repetition; Smaller and larger pitch sizes conditions were not applied with same contextual and experimental conditions.

Outcome	At least one measure of the following possibilities: Physiological responses (e.g., heart rate, blood lactate concentrations or rated of perceived exertion); Physical demands (e.g., total distance, distances covered at different speed thresholds, acceleration/ decelerations); Technical execution (e.g., passes, receptions, shots); Tactical behavior (e.g., attacking or defensive tactical principles, collective organization measures)	Other outcomes than those related to immediate physiological and physical, technical or tactical responses (e.g., fatigue tests, well-being tests).

Study design	A counterbalanced cross-over design.	Non-counterbalanced cross-over design studies.

Additional criteria	Peer reviewed, original, full-text studies written in English, Portuguese and/or Spanish.	Written in other language than those selected (English, Portuguese and/or Spanish). Reviews, letters to editors, trial registrations, proposals for protocols, editorials, book chapters, conference abstracts.

Duplicates were identified using a reference manager software (EndNote^TM^ X9, Clarivate Analytics, Philadelphia, PA, USA). Two authors (FMC and HS) independently performed screening of the title, abstract and reference list of each study to locate potentially relevant studies. Additionally, they reviewed the full version of the papers in detail to identify articles that met the selection criteria and those that were excluded. A discussion was made in the cases of discrepancies regarding the selection process with the participation of a third author (AFS).

### Information sources

Electronic databases (PubMed, PsycINFO, Scielo, Scopus, SPORT-Discus and Web of Science) were searched for relevant publications prior to the February 18, 2021. Keywords and synonyms were entered in various combinations in all fields: (“soccer” OR “football”) AND (“small-sided games” OR “conditioned games” OR “SSG” OR “drill-based games” OR “small-sided conditioned games”) AND (“pitch” OR “field”). Additionally, the reference lists of the included studies retrieved were manually searched to identify potentially eligible studies not captured by the electronic searches. Finally, an external expert in small-sided games with more than 10 publications in the last five years was contacted to verify the final list of references included in this systematic review and to indicate if there was any study that was not detected through our research.

### Extraction of data

A data extraction sheet, adapted from the Cochrane Consumers and Communication Review Group’s data extraction template [26], was used to assess inclusion requirements and subsequently tested on ten randomly selected studies (i.e., pilot testing). This process was conducted by two independent reviewers (FMC and HS). Any disagreement regarding study eligibility was resolved in a discussion between both reviewers and a third author (AFS). Full text articles excluded, with reasons, were recorded. The records were registered in a form created in Microsoft Excel (Microsoft Corporation, Readmon, WA, USA).

### Data items

Aiming to establish consistency in data analyzing and reporting, only measures that were analyzed three or more times for different articles were included. For physiological responses the following list of measures were extracted, and following this order of priority: (i) heart rate responses (e.g., absolute or relative); (ii) blood lactate concentrations; and (iii) RPE. For physical demands, the following list of measures were extracted and following this order of priority: (i) total distance covered; (ii) distance covered at different speed thresholds; (iii) accelerations and decelerations (number at different intensity thresholds); and (iv) mechanical workload measures (derived from inertial measurement unit). For technical execution the following list of measures were extracted and following this order of priority: (i) individual passes (total number, relative number considering accuracy); (ii) individual receptions (total number, relative number considering accuracy); (iii) individual shots (total number, relative number considering accuracy); and (iv) individual dribbles (total number, relative number considering accuracy). For tactical behavior the following list of measures were extracted and following this order of priority: (i) individual attacking tactical behavior; (ii) individual defensive tactical behavior; (iii) collective measure of dispersion. Tests and instruments used for measuring the outcomes were also extracted. Mean and SD for each outcome extracted in smaller and larger pitch sizes were collected. Additionally, the following information was extracted from the included studies: (i) number of participants (n), age-group (years), competitive level (e.g., elite, professional, amateur) and sex; (ii) the SSGs format (e.g., 5 vs 5; 6 vs 6), pitch size and relative area per player; (iii) regimen of intervention (work duration, work intensity, modality, relief duration, relief intensity, repetitions and series, between-set recovery).

### Assessment of methodological quality

The methodological index for non-randomized studies (MINORS) was used for assessing the methodological quality of the included studies [27]. This scale classifies twelve items of the original articles, in which a score of zero indicates the absence of a report, the score of one represents that report is inadequate and two points indicate that the report is adequate. Two of the authors (HS and MRG) independently assessed the methodological quality. Any disagreement in the rating was resolved through discussion and by a third author (FMC).

### Summary measures, synthesis of results, and publication bias

Although two studies can be used in meta-analyses [28], considering reduced sample sizes are common in the sports science literature [29], particularly SSG studies [30], analysis and interpretation of results in this systematic review and meta-analysis were only conducted in the case of at least three study groups provided mean and standard-deviation for smaller and larger pith sizes for the same measure. Means and SD for dependent variables were used to calculate effect sizes (ES; Hedge’s *g*) for each outcome in the smaller and larger pitch sizes. In case means and SDs were not available, they were obtained from 95% confidence intervals (CIs) or standard error of mean (SEM), using Cochrane’s RevMan Calculator. Data were standardized using post-intervention SD values. The random-effects model was used to account for differences between studies that might impact the SSG-based effect [31, 32]. The ES values are presented with 95% CI. Calculated ES were interpreted using the following scale: < 0.2, trivial; 0.2–0.6, small; > 0.6–1.2, moderate; > 1.2–2.0, large; > 2.0–4.0, very large; > 4.0, extremely large [33]. Heterogeneity was assessed using the *I*^2^ statistic, with values of < 25%, 25–75%, and > 75% considered to represent low, moderate, and high levels of heterogeneity, respectively [34]. The risk of bias was explored using the extended Egger’s test [35]. To adjust for publication bias, a sensitivity analysis was conducted using the trim and fill method [36], with L0 as the default estimator for the number of missing studies [37]. All analyses were carried out using the Comprehensive Meta-Analysis software (version 2; Biostat, Englewood, NJ, USA). Statistical significance was set at *p* ≤ 0.05.

### Moderator analyses

Using a random-effects model and independent computed single factor analysis, potential sources of heterogeneity likely to influence the effects of SSGs were selected *a priori*. As the responses to SSGs may be affected by the format of play, sub-group analysis considered the following the groups of formats of play [38]: (a) duels (1vs.1); (b) small formats (2vs.2, 3vs.3 and 4vs.4); (c) medium formats (5vs.5, 6vs.6, 7vs.7, 8vs.8); and (d) large formats (9vs.9, 10vs.10, 11vs.11). Additionally, information about age-group was also considered as moderator (young & youth < 23 years old since is the last category of youth in soccer; adults > 23 years old).

## RESULTS

### Study identification and selection

**FIG. 1:**
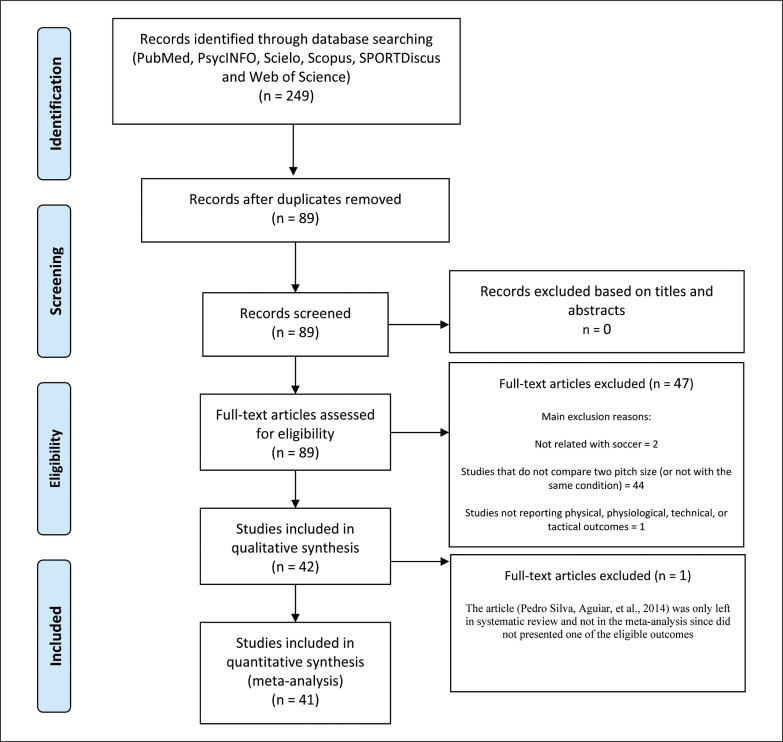
PRISMA flow diagram highlighting the selection process for studies.

The searching of databases identified an initial 249 titles. Duplicates (160 references) were subsequently removed either automatically or manually. The remaining 89 articles were screened. After reading full texts, a further 47 studies were excluded owing to a number of reasons: studies not performed in soccer, studies that not compare two pitch size (or not with the same condition), and studies not reporting physical, physiological, technical, or tactical outcomes. Therefore, 42 articles were eligible for the systematic review and 41 for the meta-analysis (Figure 1). The included articles provided mean and SD for smaller and larger pitch sizes data for at least one main outcome.

### Methodological quality

The assessment of the included studies can be found in Table 2.

**TABLE 2:** Assessment of studies methodological quality using the MINORS scale

Study	1*	2*	3*	4*	5*	6*	7*	8*	9*	10*	11*	12*	Total** (%)
[71]	1	1	1	1	0	1	1	1	-	1	1	1	90
[72]	1	0	1	1	0	0	1	1	-	1	0	1	64
[73]	1	0	1	1	0	1	1	1	-	1	0	1	72
[74]	1	0	1	1	0	1	1	1	-	1	1	1	81
[40]	1	0	1	1	0	0	1	1	-	1	0	1	64
[18]	1	1	1	1	1	0	1	1	-	1	1	1	90
[75]	1	0	1	1	0	1	1	1	-	1	0	1	72
[76]	1	1	1	1	0	0	1	1	-	1	0	1	72
[77]	1	0	1	1	0	0	1	1	-	1	1	1	72
[14]	1	0	1	1	0	1	1	1	-	1	1	1	81
[13]	1	1	1	1	0	1	1	1	-	1	1	1	90
[78]	1	0	1	1	0	1	1	1	-	1	1	1	81
[79]	1	1	1	1	0	1	1	1	-	1	1	1	90
[80]	1	1	1	1	0	0	1	1	-	1	1	1	81
[68]	1	0	1	1	0	0	1	1	-	1	.0	1	64
[81]	1	0	1	1	0	0	1	1	-	1	0	1	64
[82]	1	0	1	1	0	1	1	1	-	1	1	1	81
[69]	1	0	1	1	0	0	1	1	-	1	0	1	64
[83]	1	1	1	1	0	1	1	1	-	1	1	1	90
[84]	1	0	1	1	0	0	1	1	-	1	1	1	72
[85]	1	0	1	1	0	1	1	1	-	1	1	1	81
[61]	1	1	1	1	0	0	1	1	-	1	1	1	81
[86]	1	0	1	1	0	1	1	1	-	1	0	1	72
[87]	1	0	1	1	0	1	1	1	-	1	1	1	81
[88]	1	0	1	1	0	1	1	1	-	1	1	1	81
[39]	1	0	1	1	1	1	1	1	-	1	1	1	91
[17]	1	0	1	1	0	0	1	1	-	1	0	1	64
[89]	1	0	1	1	0	1	1	1	-	1	1	1	81
[19]	1	1	1	1	0	1	1	1	-	1	1	1	90
[90]	1	1	1	1	0	0	1	0	-	1	1	1	73
[91]	1	1	1	1	0	1	1	1	-	1	1	1	91
[92]	1	0	1	1	0	0	1	1	-	1	0	1	64
[62]	1	0	1	1	0	0	1	1	-	1	1	1	72
[93]	1	1	1	1	0	0	1	1		1	1	1	81
[66]	1	1	1	1	0	0	1	1	-	1	1	1	81
[94]	1	1	1	1	0	0	1	1	-	1	0	1	72
[95]	1	0	1	1	0	1	1	1	-	1	0	1	72
[96]	1	0	1	1	0	0	1	1	-	1	0	1	64
[20]	1	0	1	1	0	0	1	1	-	1	1	1	72
[58]	1	0	1	1	0	0	1	1	-	1	1	1	72
[97]	1	0	1	1	0	1	1	1	-	1	0	1	72
[98]	1	0	1	1	0	0	1	1	-	1	0	1	64

* : MINORS scale items number; N..1: A clearly study aimed; N.. 2: Inclusion of consecutive patients; N.. 3: Prospective collection of data; N.. 4: Endpoints appropriate to the aim of the study; N.. 5: Unbiased assessment of the study endpoint; N.. 6: Follow-up period appropriate to the aim of the study; N.. 7: Loss to follow less than 5%; N.. 8: Prospective calculation of the study size; N.. 9: An adequate control group; N..10: Contemporary groups; N.. 11: Baseline equivalence of groups; N.. 12: Adequate statistical analyses;

** : the total number of points from a ossible maximal of 24.

### Study characteristics

The characteristics of the included studies in the meta-analysis can be found in Table 3. Additionally, the details of the SSGs-interventions can be found in Table 4.

**TABLE 3:** Characteristics of the included studies and outcomes extracted.

Study	N	Mean + SD age (y)	Experience (y)	Sex	Randomization of SSGs order	Design	Variables assessed in the study and tendency	Tests or tools used	Outcome extracted
[71]	10 recreational players	31.7 ± 7.6	0.25–0.50	Male	Yes	Repeated measures	Mean HR; peak HR; RPE; HR zones; ball possession; dribbling; passes; tackles; shots. *Pitch effect:* ball possessions and unsuccessful passes were higher on a small pitch.	HR monitor; 15-point Borg scale; video cameras	Physiological responses; Technical actions

[72]	16 youth elite players	16.9 ± 0.3	-	Male	Yes	Repeated measures	Maximum velocity; mean velocity; distance covered at several velocities; accelerations. *Pitch effect:* more HI distance is covered in higher SSG.	GPS	Physical responses

[73]	9 professional soccer players	26.2 ± 3.7	5.5	Male	Yes	Repeated measures	Maximum velocity; distance/min; distance covered at several velocities; mean HR; peak HR; HR zones. *Pitch effect:* higher physical values in larger areas.	GPS; HR monitor	Physical responses; Physiological responses

[74]	20 amateur soccer players	21.0 ± 5.0	11	Male	No	Repeated measures	Peak HR; RPE; TD; maximum velocity; player load; accelerations/deceleration; change of directions. *Pitch effect:* increasing the pitch length had a greater effect compared to increasing the pitch width.	HR monitor; 10-point Borg scale; GPS	Physiological responses; Physical responses

[40]	10 male youth players	15.5 ± 0.5	7.5	Male	Yes	Repeated measures	Effective playing time; start of play; contact surface; successful actions. *Pitch effect:* increase frequency of motor actions when pitch is reduced.	Video camera	Technical actions

[18]	10 male youth players	15.5 ± 0.5	7.5	Male	Yes	Repeated measures	Effective playing time; Peak HR; HR zones; RPE; TD; distance/min; distance covered at several velocities; work-rest ratio; sprint frequency; several technical actions. *Pitch effect:* increasing the pitch size increase the effective playing time, the physical and physiological workload and the RPE, but reduce the frequency of motor actions.	HR monitor; 10-point Borg scale; GPS; vide camera	Physiological responses; Physical responses; Technical actions

[75]	19 professional players	17.1 ± 0.3	-	Male	Yes	Repeated measures	TD; HI velocity; HI accelerations; HI decelerations; HI metabolic power; Lactate; Mean HR; Peak HR; RPE. *Pitch effect:* Lower SSG elicit lower external load values than higher SSGs	GPS; HR monitor lactate portable analyser; 10-point Borg scale	Physical responses; Physiological responses

[76]	28 youth players	U13: 13.5 ± 0.3 U14: 14.3 ± 0.3	> 3	Male	Yes	Repeated measures	TD; distance covered at several velocities; player load; exertion index; work-rest ratio; Maximum velocity. *Pitch effect:* increasing pitch size elicit higher responses in both groups.	Accelerometer; GPS	Physical responses

[77]	24 youth elite players	13.3 ± 0.5	> 3	Male	Yes	Repeated measures	TD; distance covered at several velocities; player load; exertion index; work-rest ratio; Maximum velocity; Mean HR; Peak HR; RPE; HR zones. *Pitch effect:* higher pitch sizes are associated with increases in TD, work-rest ratio, player load, Peak HR and in the distance covered at 8 km/h.	GPS; HR monitor; 10-point Borg scale	Physical responses; Physiological responses

[14]	44 youth players	U12: 12.1 ± 0.4 U13: 13.3 ± 0.5	> 1	Male	No	Repeated measures	TD; distance covered at several velocities; player load; exertion index; work-rest ratio; Maximum velocity; Mean HR; Peak HR; RPE; HR zones. *Pitch effect:* higher pitch size is related with greater responses.	GPS; HR monitor; 10-point Borg scale	Physical responses; Physiological responses

[13]	28 youth players	U13: 13.5 ± 0.3 U14: 14.3 ± 0.3	> 3	Male	No	Repeated measures	Team length; width length; convex hull; stretch index; distance between centroids; length of both teams; width of both teams; convex hull of both teams; stretch index of both teams. *Pitch effect:* higher pitch size is related with greater tactical responses.	GPS	Tactical responses

[78]	24 youth players	11.8 ± 0.3	> 3	Male	Teams configuration	Repeated measures	TD; distance covered at several velocities; number of sprints; Maximum velocity; accelerations; decelerations; body impacts. *Pitch effect:* increasing pitch size elicit higher responses.	GPS	Physical responses

[79]	10 youth players	14.8 ± 0.6	> 3	Male	No	Repeated measures	TD; distance covered at several velocities; number of sprints; Maximum velocity; Mean HR; Peak HR. *Pitch effect:* increasing pitch size elicit higher responses.	GPS; HR monitor	Physical responses; Physiological responses

[80]	20 youth players	14.9 ± 0.6	> 3	Male	No	Repeated measures	TD; distance covered at several velocities; number of sprints; Maximum velocity; accelerations; decelerations; body impacts. *Pitch effect:* larger SSG demanded a higher external load in comparison with shorter SSG.	GPS	Physical responses

[68]	10 amateur players	23.4 ± 3.9	-	Male	No	Repeated measures	TD; distance covered at several velocities; number of sprints; spatial exploration index. *Pitch effect:* increasing pitch size elicit higher responses.	GPS	Physical responses; Tactical responses

[81]	10 amateur players	23.4 ± 3.9	-	Male	No	Repeated measures	Centroid; stretch index. *Pitch effect:* increasing pitch size elicit higher tactical responses.	GPS	Tactical responses

[82]	40 international players	25.3 ± 2.4	-	Male	Yes	Repeated measures	%HR reserve; Peak HR; RPE; lactate; TD, TD in sprinting; TD in HI; duels, passes, balls lost; ball possessions. *Pitch effect:* SSG elicit higher demands compared to friendly matches, except for lactate, successful passes and ball possessions.	HR monitor; GPS; semi-automatic multiple camera system; lactate portable analyser; 10-point Borg scale.	Physical responses; Physiological responses; Technical responses

[69]	10 amateur players	22.0 ± 3.0	-	Male	No	Repeated measures	Centroid; area; inter-team distance in longitudinal and lateral directions; distance of centroids. *Pitch effect:* increasing pitch size elicit greater area and distances.	LPS	Tactical responses

[83]	11 youth players	16.3 ± 0.6	> 6	Male	No	Repeated measured	Metabolic power; TD; HI demands. *Pitch effect:* increasing pitch size elicit higher responses.	GPS	Physical responses

[84]	8 amateur players	27.2 ± 3.1	12	Male	No	Repeated measured	Shots; passes; accurate passes; inaccurate passes; dribbles; interceptions; tackles. *Pitch effect:* no influence of pitch size on technical actions.	Digital cameras.	Technical responses

[85]	16 youth players	13.2 ± 0.6	> 3	Male	Yes	Repeated measures	HR; RPE: lactate. *Pitch effect:* higher physiological responses in larger pitch size.	HR monitor; lactate portable analyser; 10-point Borg scale.	Physiological responses

[61]	8 university-level players	20.0 ± 1.0	> 5	Male	Yes	Repeated measures	TD; HI distance; sprint distance; accelerations; decelerations; Peak HR; Maximum HR; pass; tackle; header; turn; interception; dribbling; shots. *Pitch effect:* increasing pitch size elicit higher responses.	GPS; HR monitor; video camera.	Physical responses; Physiological responses; Technical responses

[86]	29 junior players	18.1 ± 1.3	-	Male	No	Repeated measures	TD; Peak HR; HR zones; RPE. *Pitch effect:* increasing pitch size elicit higher physiological responses.	Video manual motion tracker; HR monitor; 10-point Borg scale	Physical responses; Physiological responses

[87]	3 youth goalkeepers	16.6 ± 0.9	7.3	Male	No	Repeated measures	Goalkeeper’s actions: Goal kick; direct free kick; indirect free kick; pass by hand; pass by foot; length; direction; area; save; deflection; clear-out; Open palm; parry; fly; 1-on-1; screen; zone intervention. *Pitch effect:* decreasing pitch size elicit higher goalkeepers’ technical responses.	Observational tool	Technical responses; Tactical responses

[88]	3 goalkeepers	24.5 ± 7.2	11	Male	No	Repeated measures	TD; spatial exploration index; predictive ellipse area; standard ellipse area; distance covered at different velocities; accelerations; decelerations. *Pitch effect:* increasing pitch size elicit lower goalkeepers’ physical responses and higher tactical responses.	GPS	Physical responses; Tactical responses

[39]	149 young players	12.0 ± 0.4	-	Male	Yes	Repeated measures	Field players and goalkeepers actions: Ball touches; Passes; Shots; TD in play; TD out play Distance covered at different velocities. *Pitch effect:* pitch size influences on physical and technical responses of field players and goalkeepers.	Semi-automated multi-camera system	Physical responses; Technical responses

[17]	8 elite players	18.0 ± 1	-	Male	Yes	Repeated measures	Mean HR; pass; receive; turn; dribble; header; tackle; interception; shot; target pass. *Pitch effect:* While pitch size does not affect physiological responses, increasing pitch size elicit higher shots and tackles.	HR monitor; video camera	Physiological responses; Technical responses

[89]	16 youth players	14.2 ± 0.6	5.5	Male	Yes	Repeated measures	Mean HR; %HRmax; RPE. *Pitch effect:* Increasing pitch size elicit higher physiological responses.	HR monitor; 10-point Borg scale	Physiological responses

[19]	48 youth players	U13 U14	-	Male	Teams’ composition	Repeated measures	TD; distance covered at different velocities; Peak HR; Mean HR. *Pitch effect:* no influence of pitch size on physical and physiological demands	GPS; HR monitor	Physiological responses; Physical responses

[90]	16 elite players	19.6 ± 2.0	5.8	Female	Yes	Repeated measures	TD; body loads, high-intensity distance. *Pitch effect:* Increasing pitch size elicit higher physical responses.	GPS	Physical responses

[91]	16 elite players	19.6 ± 2.0	5.8	Female	Yes	Repeated measures	Peak HR; Mean HR; %HRmean; HR zones; VAS scales. *Pitch effect:* Increasing pitch size elicit higher physiological responses at low intensities.	HR monitor; questionnaire	Physiological responses

[92]	10 youth players	13.0 ± 0.3	-	Male	No	Repeated measures	HR HI; passes; dribbles; possessions. *Pitch effect:* No influence in HR HI and increasing pitch size elicit higher lower possessions and higher ball touches.	HR monitor; Video camera	Physiological responses; Tecnhical responses

[62]	23 university players	22.3 ± 2.0	12.1	Male	Teams’ composition	Repeated measures	TD; distance covered at different velocities; sprints; maximum sprint speed; ball contacts; maximum passing speed; RPE. *Pitch effect:* increasing pitch size elicit higher physical responses.	GPS; 10-point Borg scale; Play Soccer system	Physical responses; Physiological responses; Technical responses

[93]	52 youth players	U11: 10.0 ± 0.7 U15: 14.0 ± 1.3 U23: 21.0 ± 1.6	1.0 3.0 6.5	Male	-	Repeated measures	TD; distance covered at different velocities; sprints; maximum sprint speed; ball contacts; maximum passing speed; RPE. *Pitch effect:* increasing pitch size elicit higher physical responses and influence on technical actions.	GPS; 10-point Borg scale; Play Soccer system	Physical responses; Physiological responses; Technical responses

[66]	148 youth players	U12: 12.5 ± 0.5 U14: 14.4 ± 0.5 U16: 16.6 ± 3.2 U18: 17.9 ± 1.0	-	Male	Teams’ composition	Repeated measures	TD; HI distance; sprints; inter-team distance, LPW-ratio, surface area, stretch indices, goalkeeper-defender distance; tactical variability. *Pitch effect:* increasing pitch size elicit higher physical responses and intra-team and inter-team distances and tactical variability.	LPS	Physical responses; Tactical responses

[94]	10 recreational players	20.1 ± 1.1	-	Male	-	Repeated measures	Mean HR; %HRmax; HR zones; TD; distance covered at different velocities; maximal speed; efforts; player load. *Pitch effect:* increasing pitch size elicit higher physiological and physical responses.	HR monitor; GPS	Physiological responses; Physical responses

[95]	20 amateur players	24.5 ± 4.1	15	Male	Yes	Repeated measures	Mean HR; Blood lactate; RPE. *Pitch effect:* increasing pitch size elicit higher physiological responses.	HR monitor; lactate portable analyser	Physiological responses

[96]	86 youth players	U10 U13	-	Male	Yes	Repeated measures	Mean HR; Peak HR; HR zones; TD; efforts; distance covered at different velocities; player load; number of technical actions; successful actions; success rate. *Pitch effect:* increasing pitch size elicit higher physical responses and lower technical involvement.	HR monitor; GPS; video camera	Physiological responses; Physical responses; Technical responses

[20]	20 youth players	16.2 ± 0.6 ^a^ 15.6 ± 0.5	6.6 6.2	Male	No	Repeated measures	Spatial distribution variability; Shannon entropy; player-to-locus distance; coefficient of variation; sample entropy. *Pitch effect:* manipulating pitch size influence on movement variability.	GPS	Tactical responses. The outcomes were not obtained for meta-analysis since none was within the information extracted, thus keeping only in the systematic review

[58]	20 youth players	16.2 ± 0.6 15.6 ± 0.5	6.6 6.2	Male	No	Repeated measures	Team separateness; effective playing space; length-width ratio; average mutual information in longitudinal direction; average mutual information in lateral direction; sample entropy of distance to nearest opponent. *Pitch effect:* manipulating pitch size influence on tactical responses.	GPS	Tactical responses

[97]	24 youth players	14.5 ± 0.5	6.1	Male	Yes	Repeated measures	Effective relative space per player; radius of free movement; spatial distribution variability; numerical relations. *Pitch effect:* manipulating pitch size influence on spatial distributions and numerical relations.	GPS	Tactical responses

[98]	15 amateur players	21.9 ± 2.0	9.9	Male	Teams’ composition	Repeated measures	Interpersonal distance attackers and defenders; distance to intercept a shot; distance to intercept a pass. *Pitch effect:* increasing pitch size elicit greater opportunities to maintain ball possessions.	Video camera	Tactical responses

[69]	10 amateur players	22.0 ± 3.0	-	Male	No	Repeated measures	Centroid; area; inter-team distance in longitudinal and lateral directions; distance of centroids. *Pitch effect:* increasing pitch size elicit greater area and distances.	LPS	Tactical responses

*Notes.* GPS: global position system; HI: high-intensity; HR: heart rate; LPS: local positioning system; RPE: rate of perceived exertion; SD: standard-deviation; SSG: small-sided games; TD: total distance; VAS: visual analogue scale.

**TABLE 4:** Characteristics of small-sided games (SSGs) in the included studies.

Study	SSG formats	Smaller pitch (length’width)	Smaller pitch (area per player -m^2^)	Larger pitch (length𡀙width)	Larger pitch (area per player -m^2^)	Larger/Smaller (m^2^)	Task conditions	Sets	Reps	Work duration	Between reps duration	Type of recovery
[71]	5vs.5 + GK 7vs.7 + GK	44 × 23 m	5 players: 101.2 m^2^ 7 players: 72.3 m^2^	57 × 30 m	5 players: m^2^ 7 players: m^2^	5 players: 1.7x 7 players: 1.7x	No throw-ins; restart the game as quickly as possible.	1	1	40 min	-	-

[72]	4vs.4	Not reported (125 m^2^)	15.6 m^2^	Not reported (300 m^2^)	37.5 m^2^	2.4x	Verbal encouragement.	1	1	8 min	-	-

[73]	6vs.6 + 1 6vs. + 1 + GK	20 × 30 m	43 m^2^	50 × 40 m	154 m^2^	3.6x	Two touch per player; verbal encouragement; restart the game as quickl as Possible.	1	4	20 min	2 min	Passive

[74]	5vs.5 + GK	40 × 25 m	100 m^2^	66 × 50 m	330 m^2^	3.3x	No offside rule.	1	4	24 min	8 min	Passive

[40]	5vs.5 + GK	32 × 23 m	73.6 m^2^	62 × 44 m	272.8 m^2^	3.7x	No offside rule; verbal encouragement.	1	3	24 min	5 min	Passive

[18]	5vs.5 + GK	32 × 23 m	73.6 m^2^	62 × 44 m	272.8 m^2^	3.7x	No offside rule; verbal encouragement.	1	3	24 min	5 min	Passive

[75]	1vs.1	20 × 10 m	100 m^2^	30 × 20 m	300 m^2^	3.0x	Verbal encouragement; restart the game as quickl as possible; players free t score from any distance; n ball touches limit.	^y^ 1 o	4	2 min	3 min	Active

[76]	7vs.7 + GK	30 × 40 m	100 m^2^	60 × 40 m	200 m^2^	2x	Restart the game as quick as possible; offside rule.	1	4	28 min	4 min	Passive

[77]	7vs.7 + GK 9vs.9 + GK 11vs.11 + GK	45 × 27 m	100 m^2^	100 × 60 m	300 m^2^	3x	Verbal encouragement; restart the game as quickl as possible.	y 1	2	24 min	5 min	Passive

[14]	7vs.7 + GK 9vs.9 + GK 11vs.11 + GK	45 × 27 m	100 m^2^	100 × 60 m	300 m^2^	3x	Verbal encouragement; restart the game as quickl as possible.	y 1	2	24 min	5 min	Passive

[13]	7vs.7 + GK	30 × 40 m	100 m^2^	60 × 40 m	200 m^2^	2x	Restart the game as quick as possible; offside rule.	1	4	28 min	4 min	Passive

[78]	6vs.6	22 × 13 m	25 m^2^	39 × 23	75 m^2^	3x	Restart the game as quick as possible; verbal encouragement; offside rul in some configurations.	l	1	6 min	-	-

[79]	5vs.5 + GK	38 × 26 m	100 m^2^	53 × 37 m	200 m^2^	2x	Restart the game as quick as possible; verbal encouragement.	1	6	4 min	2 min	Active
									4	6 min		

[80]	5vs.5 + GK	38 × 26 m	100 m^2^	53 × 37 m	200 m^2^	2x	Restart the game as quick as possible; verbal encouragement; offside rule.	1	4	6 min	2 min	Active

[68]	11vs.11 + GK	54 × 68 m	167 m^2^	108 × 68 m	334 m^2^	2x	Official game rules	1	1	30 min	-	-

[81]	11vs.11 + GK	54 × 68 m	167 m^2^	108 × 68 m	334 m^2^	2x	Official game rules.	1	1	30 min	-	-

[82]	11vs.11 + GK	30 × 20 m	75 m^2^	100 × 60 m	273 m^2^	3.6x	Touch limitation (1, 2 or free).	1	4	16 min	3 min	Passive

[69]	4vs.4 + GK	24 × 16 m	38.4 m^2^	30 × 20 m	60 m^2^	1.6x	No offside rule; GK had 2-touch play; outfield players had to avoid long-range shots.	1	1	8 min	8 min	-

[83]	5vs.5 + GK	39 × 25 m	81 m^2^	78 × 50 m	325 m^2^	4x	No offside rule.	1	1	35 min	-	-

[84]	4vs.4	34 × 26 m	111 m^2^	40 × 30 m	150 m^2^	1.4x	Miniature goals; Restart the game as quickly as possible; no offside rule.	1	3	18 min	5 min	-

[85]	4vs.4	10 × 15 m	19 m^2^	20 × 25 m	62.5 m^2^	3.3x	Restart the game as quickly as possible; verbal encouragement; free touches.	1	4	16 min	2 min	Passive

[61]	5vs.5 + GK	30 × 20 m	60 m^2^	50 × 40 m	200 m^2^	3.3x	Tournament scenario; restart the game as quickly as possible; verbal encouragement.	1	4	16 min	3 min	-

[86]	5vs.5 + GK 5vs.5	28 × 20 m	56 m^2^	42 × 30 m	126 m^2^	2.3x	Balls were disposed around the game areas.	1	3	12 min	3 min	Active

[87]	5vs.5 + GK	32 × 23 m	73.6 m^2^	62 × 44 m	272.8 m^2^	3.7x	Goalkeepers restart the game as quickly as possible.	1	3	24 min	5 min	Passive

[88]	5vs.5 + GK	32 × 23 m	73.6 m^2^	62 × 44 m	272.8 m^2^	3.7x	Goalkeepers restart the game as quickly as possible.	1	3	24 min	5 min	Passive

[39]	8vs.8 + GK	68 × 47 m	199.75 m^2^	75 × 47 m	220.31 m^2^	1.1x	Balls were disposed around the game areas; verbal encouragement.	1	1	30 min	-	-

[17]	5vs.5 + GK	30 × 20 m	60 m^2^	50 × 40 m	200 m^2^	3.3x	Balls were disposed around the game areas; verbal encouragement.	1	4	16 min	2 min	Active

[89]	3vs.3 + 4	20 × 15 m	50 m^2^	30 × 20 m	100 m^2^	2.0x	Balls were disposed around the game areas; verbal encouragement.	1	4	12 min	2 min	Passive
	4vs.4 + 4	20 × 20 m	50 m^2^	32 × 25 m	100 m^2^			1	4	16 min	2 min	

[19]	3vs.3 + GK + 1	36 × 27 m	138.9 m^2^	40 × 29	165.7 m^2^	1.2x	Offside rule; balls were disposed around the game areas; verbal encouragement; not technical and tactical instructions.	1	4	16 min	4 min	Passive

[90]	4vs.4	20 × 20 m	50 m^2^	28.3 × 28.3 m	100 m^2^	2x	Balls were disposed around the game areas; verbal encouragement.	1	3	4 min	10 min	Active

[91]	4vs.4	20 × 20 m	50 m^2^	28.3 × 28.3 m	100 m^2^	2x	Balls were disposed around the game areas; verbal encouragement.	1	3	4 min	10 min	Active

[92]	5vs.5	30 × 20 m	60 m^2^	51 × 34 m	173.4 m^2^	2.9x	Stop the ball with their foot backside to the limits of the pitch to score; balls were disposed around the game areas.	1	4	16 min	1 min	Passive

[62]	4vs.3	20 × 15 m	42.9 m^2^	30 × 25 m	107.1 m^2^	2.5x	Coach did not intervene; Balls were disposed around the game areas.	1	4	16 min	4 min	Active
	4vs.4		37.5 m^2^		93.75 m^2^	2.5x						
	4vs.5		33.3 m^2^		83.3 m^2^	2.5x						

[93]	4vs.4	20 × 15 m	37.5 m^2^	30 × 25 m	93.75 m^2^	2.5x	Coach did not intervene; Balls were disposed around the game areas.	1	4	16 min	4 min	Active

[66]	4vs.4 + GK	40 × 30 m	120 m^2^	68 × 47 m	320 m^2^	2.7x	Coach players similar to match; offside rule in large pitch; no offside rule in small pitch.	1	5	20 min	4 min	-

[94]	4vs.4 + GK	37 × 17 m	60 m^2^	40 × 20 m	80 m^2^	1.3x	No verbal encouragement; Balls were disposed around the game areas; one referee.	1	2	40 min	5 min	Passive

[95]	3vs.3	12 × 20 m	40 m^2^	18 × 30 m	90 m^2^	2.3x	With and without coach encouragement.	1	3	12 min	3 min	Active
	4vs.4	16 × 24 m	48 m^2^	24 × 36 m	108 m^2^	2.3x						
	5vs.5	20 × 28 m	56 m^2^	30 × 42 m	126 m^2^	2.3x						
	6vs.6	24 × 32 m	64 m^2^	36 × 48 m	144 m^2^	2.3x						

[96]	5vs.5	30 × 40 m	120 m^2^	105 × 68 m	325 m^2^	2.7x	Smaller goals or normal goals.	1	1	20 min	-	-
	8vs.8											

[20]	4vs.4 + GK	23.8 × 36.8 m	88 m^2^	37.4 × 57.8 m	216 m^2^	2.5x	Not allowed passing to the goalkeeper; coach did not intervene.	1	3	7 min	7 min	Active

[58]	4vs.4 + GK	36.8 × 23.8 m	87.6 m^2^	57.8 × 37.4 m	216.2 m^2^	2.5x	Not allowed passing to the goalkeeper.	1	3	7 min	7 min	Active

[97]	6vs.6	46.7 × 30.3 m	118 m^2^	52.9 × 34.4 m	152 m^2^	1.3x	Scoring zone; balls were disposed around the game areas; coach did not intervene.	1	3	18 min	4 min	Passive

[98]	5vs.5	28 × 14 m	39.2 m^2^	52 × 26 m	135.2 m^2^	3.4x	Small goals.	1	2	10 min	5 min	-

[69]	4vs.4 + GK	24 × 16 m	38.4 m^2^	30 × 20 m	60 m^2^	1.6x	No offside rule; GK had 2-touch play; outfield players had to avoid long-range shots.	1	1	8 min	8 min	-

*Notes.* GK: goalkeepers;

### Smaller vs. larger pitch sizes during SSG: effects on physiological responses

A summary of the included studies and results of physiological responses (HR and RPE) reported in smaller and larger SSGs are provided in Table 5.

**TABLE 5:** Summary of the included studies and results of physiological responses in smaller and larger pitch sizes.

Study	Format	Age category	N	Variable	Smaller Mean ± SD	Larger Mean ± SD	Larger-Smaller (%)	Tendency of change	Included in the meta-analysis
[71]	5vs.5 + GK	Adults	10	HR	164.3 ± 11.9	167.0 ± 13.2	1.9	Increase in larger pitch size	Yes

[71]	7vs.7 + GK	Adults	10	HR	161.2 ± 12.9	163.5 ± 12.8	1.4	Increase in larger pitch size	Yes

[71]	5vs.5 + GK	Adults	10	RPE	12.4 ± 1.2	13.2 ± 1.9	6.5	Increase in larger pitch size	Yes

[71]	7vs.7 + GK	Adults	10	RPE	12.3 ± 0.9	12.8 ± 1.2	4.1	Increase in larger pitch size	Yes

[73]	6vs.6 + 1	Adults	9	HR	86.7 ± 7.7	89.1 ± 4.6	2.8	Increase in larger pitch size	Yes

[74]	5vs.5 + GK	Adults	20	HR	83.4 ± 5.1	86.5 ± 4.5	3.7	Increase in larger pitch size	Yes

[74]	5vs.5 + GK	Adults	20	RPE	3.8 ± 1.5	6.6 ± 1.2	84.8	Increase in larger pitch size	Yes

[18]	5vs.5 + GK	Youth ^f^	10	HR	86.0 ± 5.8	88.9 ± 3.9	4.5	Increase in larger pitch size	Yes

[18]	5vs.5 + GK	Youth ^f^	10	RPE	5.7 ± 1.0	6.7 ± 0.8	17.5	Increase in larger pitch size	Yes

[75]	1vs.1	Youth ^g^	19	HR	157 ± 8	169 ± 6	7.6	Increase in larger pitch size	Yes

[75]	1vs.1	Youth ^g^	19	RPE	5.1 ± 1.6	8 ± 1	56.9	Increase in larger pitch size	Yes

[77]	7vs.7 + GK	Youth ^c^	24	HR	82 ± 3	88 ± 6	7.3	Increase in larger pitch size	Yes

[77]	7vs.7 + GK	Youth ^c^	24	RPE	267.1 ± 47.5	299.9 ± 41.3	12.3	Increase in larger pitch size	Yes

[77]	9vs.9 + GK	Youth ^c^	24	HR	83 ± 6	85 ± 6	2.4	Increase in larger pitch size	Yes

[77]	9vs.9 + GK	Youth ^c^	24	RPE	233.4 ± 28.7	270.9 ± 25.8	16.1	Increase in larger pitch size	Yes

[77]	11vs.11 + GK	Youth ^c^	24	HR	81 ± 4	88 ± 4	8.6	Increase in larger pitch size	Yes

[77]	11vs.11 + GK	Youth ^c^	24	RPE	228.6 ± 49.3	306.1 ± 39.3	33.9	Increase in larger pitch size	Yes

[14]	7vs.7 + GK	Youth ^b^	22	HR	85.3 ± 7.1	88.3 ± 3.3	3.5	Increase in larger pitch size	Yes

[14]	7vs.7 + GK	Youth ^b^	22	RPE	283 ± 26	297 ± 25	4.9	Increase in larger pitch size	Yes

[14]	9vs.9 + GK	Youth ^b^	22	HR	84.7 ± 2.9	83.3 ± 4.5	-1.7	Decrease in larger pitch size	Yes

[14]	9vs.9 + GK	Youth ^b^	22	RPE	297 ± 35	310 ± 35	4.4	Increase in larger pitch size	Yes

[14]	11vs.11 + GK	Youth ^b^	22	HR	80.0 ± 5.5	83.1 ± 5.0	3.9	Increase in larger pitch size	Yes

[14]	11vs.11 + GK	Youth ^b^	22	RPE	257 ± 55	285 ± 41	10.9	Increase in larger pitch size	Yes

[14]	7vs.7 + GK	Youth ^c^	22	HR	81.8 ± 10.5	87.4 ± 4.4	3.5	Increase in larger pitch size	Yes

[14]	7vs.7 + GK	Youth ^c^	22	RPE	267 ± 47.5	300 ± 41	12.4	Increase in larger pitch size	Yes

[14]	9vs.9 + GK	Youth ^c^	22	HR	82.6 ± 4.7	84.7 ± 2.6	2.54	Increase in larger pitch size	Yes

[14]	9vs.9 + GK	Youth ^c^	22	RPE	297 ± 35	271 ± 26	-8.8	Decrease in larger pitch size	Yes

[14]	11vs. 11 + GK	Youth ^c^	22	HR	80.8 ± 2.8	87.6 ± 3.1	8.4	Increase in larger pitch size	Yes

[14]	11vs.11 + GK	Youth ^c^	22	RPE	257 ± 55	306 ± 39	19.1	Increase in larger pitch size	Yes

[79]	5vs.5 + GK	Youth ^e^	10	HR	180 ± 8	180 ± 8	0.0	No differences	Yes

[82]	11vs.11 + GK	Adults	40	HR	84.7 ± 2.7	83.2 ± 2.6	-1.8	Decrease in larger pitch size	Yes

[82]	11vs.11 + GK	Adults	40	RPE	7.3 ± 0.6	7.4 ± 0.5	1.4	Increase in larger pitch size	Yes

[85]	4vs.4 (SB)	Youth ^d^	16	HR	166.9 ± 3.1	174.9 ± 3.2	4.8	Increase in larger pitch size	Yes

[85]	4vs.4 (SB)	Youth ^d^	16	RPE	6.3 ± 0.9	7.1 ± 0.9	12.7	Increase in larger pitch size	Yes

[85]	4vs.4 (SG)	Youth ^d^	16	HR	163.9 ± 3.2	170.9 ± 2.7	4.3	Increase in larger pitch size	Yes

[85]	4vs.4 (SG)	Youth ^d^	16	RPE	5.8 ± 0.9	6.8 ± 0.8	17.2	Increase in larger pitch size	Yes

[61]	5vs.5 + GK	Youth ^i^	8	HR	164 ± 14	168 ± 17	2.4	Increase in larger pitch size	Yes

[86]	5vs.5 + GK	Youth ^h^	29	HR	169.3 ± 6.2	176.4 ± 7.5	4.2	Increase in larger pitch size	Yes

[86]	5vs.5 + GK	Youth ^h^	29	RPE	4.9 ± 1.3	7.5 ± 1.8	53.1	Increase in larger pitch size	Yes

[86]	5vs.5	Youth ^h^	29	HR	174.2 ± 6.5	177.1 ± 5.8	1.7	Increase in larger pitch size	Yes

[86]	5vs.5	Youth ^h^	29	RPE	4.7 ± 1.1	7.1 ± 1.1	51.1	Increase in larger pitch size	Yes

[17]	5vs.5 + GK	Youth ^h^	8	HR	175 ± 9	169 ± 6	-3.4	Decrease in larger pitch size	Yes

[89]	3vs.3 + 4	Youth ^d^	16	HR	176.3 ± 2.5	184.2 ± 6.5	4.5	Increase in larger pitch size	Yes

[89]	4vs.4 + 4	Youth ^d^	16	HR	175.0 ± 7.7	183.5 ± 8.4	4.9	Increase in larger pitch size	Yes

[19]	3vs.3 + GK + 1	Youth ^c^	24	HR	168.0 ± 10.7	166.3 ± 11.9	-1.0	Increase in smaller pitch size	Yes

[19]	3vs.3 + GK + 1	Youth ^d^	24	HR	164.1 ± 12.5	168.9 ± 11.2	2.9	Increase in larger pitch size	Yes

[91]	4vs.4	Youth ^i^	16	HR	169.4 ± 12.1	169.3 ± 11.6	-0.1	Decrease in larger pitch size	Yes

[91]	4vs.4	Youth ^i^	16	HR	163.7 ± 10.9	164.4 ± 9.7	0.4	Increase in larger pitch size	Yes

[91]	4vs.4	Youth ^i^	16	HR	160.1 ± 8.8	165.5 ± 8.7	3.4	Increase in larger pitch size	Yes

[92]	5vs.5	Youth ^c^	10	HR	85 ± 4	85 ± 5	0.0	No differences	Yes

[62]	4vs.3	Youth ^i^	20	RPE	4.0 ± 0.5	3.8 ± 1.1	-5.0	Decrease in larger pitch size	Yes

[62]	4vs.4	Youth ^i^	20	RPE	4.3 ± 0.8	4.0 ± 1.1	-7.0	No differences	Yes

[62]	4vs.5	Youth ^i^	20	RPE	4.5 ± 0.8	5.0 ± 1.1	11.1	Increase in larger pitch size	Yes

[62]	4vs.2 + 1	Youth ^i^	20	RPE	4.5 ± 0.8	5.3 ± 0.8	17.8	Increase in larger pitch size	Yes

[62]	4vs.2 + 2	Youth ^i^	20	RPE	3.8 ± 0.8	5.3 ± 1.3	39.5	Increase in larger pitch size	Yes

[62]	4vs.2 + 3	Youth ^i^	20	RPE	3.5 ± 1.1	3.3 ± 1.3	-5.7	No diferences	Yes

[93]	4vs.4	Youth ^a^	16	RPE	3.9 ± 1.1	3.5 ± 1.0	-11.4	Decrease in larger pitch size	Yes

[93]	4vs.4	Youth ^e^	18	RPE	4.3 ± 1.1	4.6 ± 1.0	7.0	Increase in larger pitch size	Yes

[93]	4vs.4	Youth ^i^	18	RPE	4.7 ± 0.9	4.9 ± 0.9	4.3	Increase in larger pitch size	Yes

[94]	4vs.4 + GK	Youth ^i^	10	HR	160 ± 10	167 ± 9	4.4	Increase in larger pitch size	Yes

[95]	3vs.3	Adults	20	HR	89.5 ± 2.9	90.9 ± 2.0	1.4	Increase in larger pitch size	Yes

[95]	4vs.4	Adults	20	HR	88.7 ± 2.0	89.7 ± 1.8	1.0	Increase in larger pitch size	Yes

[95]	5vs.5	Adults	20	HR	87.8 ± 3.6	88.8 ± 2.3	1.0	Increase in larger pitch size	Yes

[95]	6vs.6	Adults	20	HR	86.4 ± 2.0	86.9 ± 2.4	0.5	Increase in larger pitch size	Yes

[95]	3vs.3	Adults	20	RPE	8.1 ± 0.6	8.5 ± 0.4	4.9	Increase in larger pitch size	Yes

[95]	4vs.4	Adults	20	RPE	7.6 ± 0.5	8.1 ± 0.-5	6.6	Increase in larger pitch size	Yes

[95]	5vs.5	Adults	20	RPE	7.2 ± 0.9	7.5 ± 0.6	4.2	Increase in larger pitch size	Yes

[95]	6vs.6	Adults	20	RPE	6.8 ± 0.6	7.2 ± 0.8	5.9	Increase in larger pitch size	Yes

[95]	3vs.3	Adults	20	HR	87.6 ± 1.7	89.1 ± 1.8	1.5	Increase in larger pitch size	Yes

[95]	4vs.4	Adults	20	HR	86.5 ± 3.4	87.2 ± 2.8	0.7	Increase in larger pitch size	Yes

[95]	5vs.5	Adults	20	HR	86.0 ± 4.0	86.9 ± 3.2	0.9	Increase in larger pitch size	Yes

[95]	6vs.6	Adults	20	HR	83.8 ± 5.0	85.0 ± 3.6	1.2	Increase in larger pitch size	Yes

[95]	3vs.3	Adults	20	RPE	6.6 ± 0.4	7.2 ± 0.7	0.6	Increase in larger pitch size	Yes

[95]	4vs.4	Adults	20	RPE	6.3 ± 0.5	6.8 ± 0.5	0.5	Increase in larger pitch size	Yes

[95]	5vs.5	Adults	20	RPE	5.9 ± 0.7	6.2 ± 0.6	0.3	Increase in larger pitch size	Yes

[95]	6vs.6	Adults	20	RPE	4.8 ± 0.9	5.9 ± 0.5	1.1	Increase in larger pitch size	Yes

[96]	5vs.5	Youth ^a^	45	HR	174 ± 10	168 ± 12	-3.4	Decrease in larger pitch size	Yes
	8vs.8								

[96]	8vs.8	Youth ^c^	41	HR	170 ± 10	171 ± 12	0.6	Increase in larger pitch size	Yes
	11vs.11								

SD: standard-deviation; HR: heart rate; RPE: rate of perceived exertion; SB: stop-ball; SG: small-goals; NR: data not reported; %: percentage of difference; a: Under-11 or below group; b: Under-12; group; c: Under-13 group; d: Under-14 group; e; under-15 group; f: under-16 group; g: under-17 group; h: under-18 group; i: under-23 group.

**FIG. 2:**
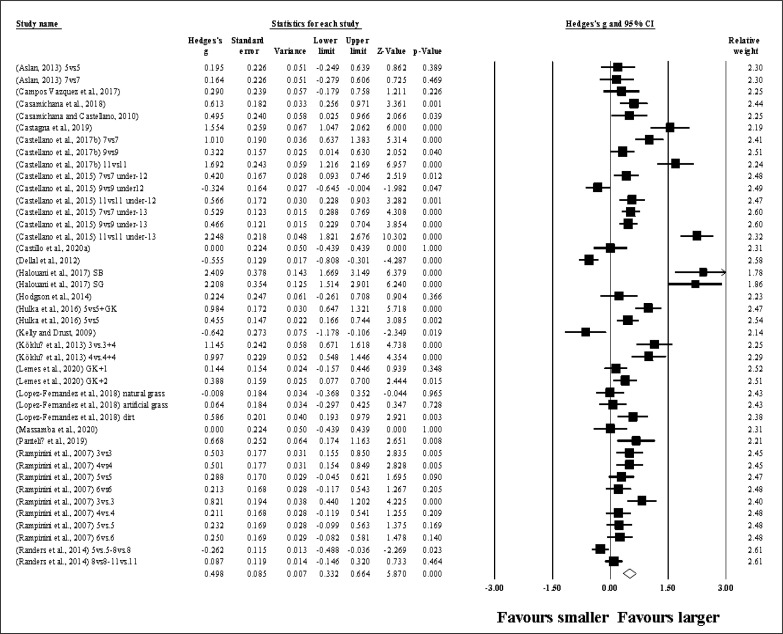
Forest plot of changes in heart rate, in soccer players participating in small-sided games using smaller compared to larger pitch sizes. Values shown are effect sizes (Hedges’s g) with 95% confidence intervals (CI). The size of the plotted squares reflects the statistical relative weight of the study. The withe diamond reflects the overall result.

Fourty-two study groups provided data for HR, involving 42 smaller and 42 larger pitch sizes being compared (pooled *n* = 898). Results (Figure 2) showed that SSGs played at larger pitches induced greater HR compared to smaller pitches (ES = 0.50, small; 95% CI = 0.33 to 0.66; *p <* 0.001; *I*^2^ = 89.1%; Egger’s test *p <* 0.001, with a corrected value of ES = 0.67, 95% CI = 0.48 to 0.86; supplementary Figure 1).

**FIG. 3:**
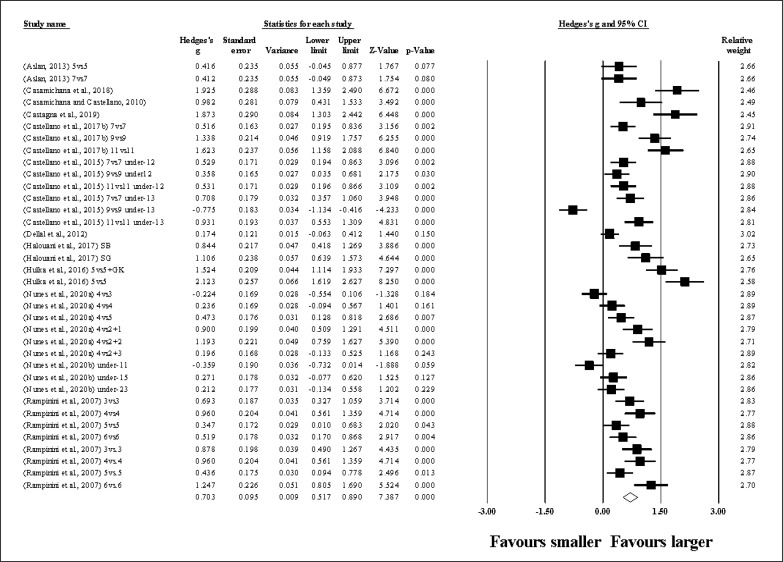
Forest plot of changes in rating of perceived exertion, in soccer players participating in small-sided games using smaller compared to larger pitch sizes. Values shown are effect sizes (Hedges’s g) with 95% confidence intervals (CI). The size of the plotted squares reflects the statistical relative weight of the study. The withe diamond reflects the overall result.

Thirty-six study groups provided data for RPE, involving 32 smaller and 32 larger pitch sizes being compared (pooled *n* = 735). Results (Figure 3) showed that SSGs played at larger pitches induced greater RPE compared to smaller pitches (ES = 0.70, moderate; 95% CI = 0.52 to 0.89; *p <* 0.001; *I*^2^ = 88.6%; Egger’s test *p <* 0.001, with a corrected value equal to the observed value; supplementary Figure 2).

### Moderator analyses for HR and RPE

The HR was greater at larger compared to smaller pitches when SSGs were played with either small (14 study groups; ES = 0.55; p < 0.001; *I*^2^ = 84.9%), medium (19 study groups; ES = 0.30; p = 0.001; I^2^ = 79.2%) and large (8 study groups; ES = 0.69; p = 0.048; *I*^2^ = 96.1%) formats, although without significant subgroup difference between formats (*p* = 0.056).

The HR was greater at larger compared to smaller pitches when SSGs were played by young/youth (29 study groups; ES = 0.60; p < 0.001; *I*^2^ = 90.9%) and adult players (13 study groups; ES = 0.28; p = 0.009; *I*^2^ = 78.4%), with significant sub-group difference according to the age of the soccer players (*p* = 0.037).

The RPE was greater at larger compared to smaller pitches when SSGs were played with either small (13 study groups; ES = 0.48; p < 0.001; *I*^2^ = 84.2%), medium (15 study groups; ES = 0.89; p < 0.001; I^2^ = 83.5%) and large (7 study groups; ES = 0.59; p = 0.03; *I*^2^ = 93.9%) formats, although without significant subgroup difference between formats (*p* = 0.088).

The RPE was greater at larger compared to smaller pitches when SSGs were played by young/youth (24 study groups; ES = 0.69; p < 0.001; *I*^2^ = 90.9%) and adult players (12 study groups; ES = 0.72; p < 0.001; *I*^2^ = 79.9%), without significant sub-group difference according to the age of the soccer players (*p* = 0.895).

### Smaller vs. larger pitch sizes during SSG: effects on physical demands

A summary of the included studies and results of physical demands (TD, HSR, ACC and DEC) reported in smaller and larger SSGs are provided in Table 6.

**TABLE 6:** Summary of the included studies and results of physical demands in smaller and larger pitch sizes.

Study	Format	Age category	N	Variable	Smaller Mean ± SD	Larger Mean ± SD	Larger-Smaller (%)	Tendency of change	Included in the meta-analysis
[72]	4vs.4	Youth ^g^	16	TD	1000 ± 131	1095 ± 89	9.5	Increase in larger pitch size	Yes

[72]	4vs.4	Youth ^g^	16	HSR	19.3 ± 14.9	64.9 ± 24.8	45.6	Increase in larger pitch size	Yes

[72]	4vs.4	Youth ^g^	16	ACC	12.2 ± 5.5	10.5 ± 3.8	-13.9	Decrease in larger pitch size	Yes

[73]	6vs.6 + 1	Adults	9	TD	93.8 ± 11.6	103.4 ± 11.2	10.2	Increase in larger pitch size	Yes

[73]	6vs.6 + 1	Adults	9	HSR	46.9 ± 22.1	492.2 ± 181.3	949	Increase in larger pitch size	Yes

[74]	5vs.5 + GK	Adults	20	TD	101.2 ± 11.8	131.4 ± 14.4	29.8	Increase in larger pitch size	Yes

[74]	5vs.5 + GK	Adults	20	ACC	3.8 ± 3.3	2.3 ± 2.4	-39.5	Decrease in larger pitch size	Yes

[74]	5vs.5 + GK	Adults	20	DEC	4.5 ± 4.1	1.8 ± 2.0	-60	Decrease in larger pitch size	Yes

[18]	5vs.5 + GK	Youth ^f^	10	TD	87.0 ± 4.6	125.0 ± 6.2	43.7	Increase in larger pitch size	Yes

[18]	5vs.5 + GK	Youth ^f^	10	HSR	4.9 ± 5.5	74.2 ± 58.9	1414.3	Increase in larger pitch size	Yes

[75]	1vs.1	Youth ^g^	19	TD	378 ± 46	601 ± 54	58.9	Increase in larger pitch size	Yes

[75]	1vs.1	Youth ^g^	19	HSR	15 ± 9	146 ± 25	873.3	Increase in larger pitch size	Yes

[75]	1vs.1	Youth ^g^	19	ACC	82 ± 16	145 ± 14	76.8	Increase in larger pitch size	Yes

[75]	1vs.1	Youth ^g^	19	DEC	34 ± 6	69 ± 6	102.9	Increase in larger pitch size	Yes

[76]	7vs.7 + GK	Youth ^c^	14	TD	663.9 ± 76.6	819.7 ± 106.5	23.5	Increase in larger pitch size	Yes

[76]	7vs.7 + GK	Youth ^c^	14	HSR	7.7 ± 8.4	38.1 ± 38.2	394.8	Increase in larger pitch size	Yes

[76]	7vs.7 + GK	Youth ^d^	14	TD	670.9 ± 67.9	871.0 ± 81.9	29.8	Increase in larger pitch size	Yes

[76]	7vs.7 + GK	Youth ^d^	14	HSR	6.5 ± 8.1	59.9 ± 43.5	821.5	Increase in larger pitch size	Yes

[77]	7vs.7 + GK	Youth ^d^	24	TD	1816 ± 155	2307 ± 212	27.1	Increase in larger pitch size	Yes

[77]	7vs.7 + GK	Youth ^d^	24	HSR	48 ± 31	202 ± 78	320.8	Increase in larger pitch size	Yes

[77]	9vs.9 + GK	Youth ^d^	24	TD	1845 ± 141	2250 ± 107	21.9	Increase in larger pitch size	Yes

[77]	9vs.9 + GK	Youth ^d^	24	HSR	70 ± 32	164 ± 41	134.3	Increase in larger pitch size	Yes

[77]	11vs.11 + GK	Youth ^d^	24	TD	1766 ± 181	2314 ± 134	31.1	Increase in larger pitch size	Yes

[77]	11vs.11 + GK	Youth ^d^	24	HSR	62 ± 43	200 ± 105	222.6	Increase in larger pitch size	Yes

[14]	7vs.7 + GK	Youth ^b^	22	TD	1718 ± 150	2186 ± 90	27.2	Increase in larger pitch size	Yes

[14]	7vs.7 + GK	Youth ^b^	22	HSR	72 ±	199 ±	176.4	Increase in larger pitch size	No Reason: SD not reported

[14]	9vs.9 + GK	Youth ^b^	22	TD	1867 ± 126	2159 ± 183	15.6	Increase in larger pitch size	Yes

[14]	9vs.9 + GK	Youth ^b^	22	HSR	89 ±	197 ±	121.3	Increase in larger pitch size	No Reason: no reported SD

[14]	11vs. 11 + GK	Youth ^b^	22	TD	1844 ± 254	2168 ± 127	17.6	Increase in larger pitch size	Yes

[14]	11vs.11 + GK	Youth ^b^	22	HSR	109 ±	236 ±	116.5	Increase in larger pitch size	No Reason: no reported SD

[14]	7vs.7 + GK	Youth ^c^	22	TD	1816 ± 155	2307 ± 212	34.3	Increase in larger pitch size	Yes

[14]	7vs.7 + GK	Youth ^c^	22	HSR	55 ± NR	218 ± NR	296.4	Increase in larger pitch size	No Reason: SD not reported

[14]	9vs.9 + GK	Youth ^c^	22	TD	1845 ± 141	2250 ± 107	20.5	Increase in larger pitch size	Yes

[14]	9vs.9 + GK	Youth ^c^	22	HSR	91 ± NR	181 ± NR	98.9	Increase in larger pitch size	No Reason: no reported SD

[14]	11vs.11 + GK	Youth ^c^	22	TD	1766 ± 181	2314 ± 134	25.5	Increase in larger pitch size	Yes

[14]	11vs.11 + GK	Youth ^c^	22	HSR	72 ± NR	218 ± NR	202.8	Increase in larger pitch size	No Reason: no reported SD

[78]	6vs.6	Youth ^b^	24	TD	466.1 ± 61.1	579.0 ± 90.1	24.2	Increase in larger pitch size	Yes

[78]	6vs.6	Youth ^b^	24	HSR	3.4 ± 5.3	31.2 ± 26.1	817.6	Increase in larger pitch size	Yes

[79]	5vs.5 + GK	Youth ^e^	10	TD	2254 ± 241	2603 ± 261	15.5	Increase in larger pitch size	Yes

[79]	5vs.5 + GK	Youth ^e^	19	HSR	4 ± 9	23 ± 26	475	Increase in larger pitch size	Yes

[80]	5vs.5 + GK	Youth ^e^	20	TD	2223 ± 248	2629 ± 227	18.3	Increase in larger pitch size	Yes

[80]	5vs.5 + GK	Youth ^e^	20	HSR	3 ± 4	28 ± 21	984.3	Increase in larger pitch size	Yes

[80]	5vs.5 + GK	Youth ^e^	20	ACC	409 ± 47	403 ± 57	-1.6	Decrease in larger pitch size	Yes

[80]	5vs.5 + GK	Youth ^e^	20	DEC	353 ± 52	361 ± 53	2.3	Increase in larger pitch size	Yes

[68]	11vs.11 + GK	Adults	10	TD	2511.2 ± 279.7	3136.6 ± 323.8	24.9	Increase in larger pitch size	Yes

[68]	11vs.11 + GK	Adults	10	HSR	93.6 ± 43.5	256.2 ± 76.2	173.7	Increase in larger pitch size	Yes

[82]	11vs.11 + GK	Adults	40	TD	2664 ± 237	11173 ± 524	319.4	Increase in larger pitch size	Yes

[82]	11vs.11 + GK	Adults	40	HSR	353 ± 59.1	483 ± 71.2	36.8	Increase in larger pitch size	Yes

[83]	5vs.5 + GK	Youth ^g^	11	TD	3067 ± 383	4068 ± 332	32.6	Increase in larger pitch size	Yes

[83]	5vs.5 + GK	Youth ^g^	11	HSR	98 ± 47	538 ± 157	448.9	Increase in larger pitch size	Yes

[61]	5vs.5 + GK	Youth ^i^	8	TD	1532 ± 145	1934 ± 133	26.2	Increase in larger pitch size	Yes

[61]	5vs.5 + GK	Youth ^i^	8	HSR	0 ± 0	61 ± 47	6100	Increase in larger pitch size	Yes

[61]	5vs.5 + GK	Youth ^i^	8	ACC	230 ± 111	327 ± 70	42.2	Increase in larger pitch size	Yes

[61]	5vs.5 + GK	Youth ^i^	8	DEC	198 ± 89	298 ± 68	50.5	Increase in larger pitch size	Yes

[86]	5vs.5 + GK	Youth ^h^	29	TD	372.4 ± 13.8	496.8 ± 26.1	33.4	Increase in larger pitch size	Yes

[86]	5vs.5	Youth ^h^	29	TD	355.6 ± 17.0	488.7 ± 26.7	37.4	Increase in larger pitch size	Yes

[88]	5vs.5 + GK	Adults	3	TD	445.1 ± 44.3	255.2 ± 25.9	-42.7	Decrease in larger pitch size	Yes

[88]	5vs.5 + GK	Adults	3	HSR	0	1.6 ± 2.1	-	Increase in larger pitch size	Yes

[88]	5vs.5 + GK	Adults	3	ACC	5.5 ± 3.9	2.7 ± 1.9	-50.9	Decrease in larger pitch size	Yes

[88]	5vs.5 + GK	Adults	3	DEC	4.2 ± 2.9	2.5 ± 1.6	-40.5	Decrease in larger pitch size	Yes

[39]	8vs.8 + GK	Youth ^b^	149	TD	2420.9 ± 215.7	2494.9 ± 203.3	3.1	Increase in larger pitch size	Yes

[39]	8vs.8 + G K	Youth ^b^	149	HSR	1108.8 ± 492.8	924.0 ± 369.6	-16.7	Decrease in larger pitch size	Yes

[19]	3vs.3 + GK + 1	Youth ^c^	48	TD	447.7 ± 45.3	457.8 ± 49.9	2.3	Increase in larger pitch size	Yes

[19]	3vs.3 + GK + 1	Youth ^d^	48	HSR	13.5 ± 0.4	15.0 ± 0.5	11.1	Increase in larger pitch size	Yes

[90]	4vs.4	Youth ^i^	16	TD	399.0 ± 33.4	458.6 ± 52.0	14.9	Increase in larger pitch size	Yes

[90]	4vs.4	Youth ^i^	16	HSR	21.0 ± 11.3	55.1 ± 31.3	162.4	Increase in larger pitch size	Yes

[62]	4vs.3	Youth ^i^	20	HSR	1.0 ± 1.1	5.8 ± 5.1	480	Increase in larger pitch size	Yes

[62]	4vs.4	Youth ^i^	20	HSR	2.0 ± 2.1	9.3 ± 8.3	365	Increase in larger pitch size	Yes

[62]	4vs.5	Youth ^i^	20	HSR	1.5 ± 1.3	10.8 ± 6.7	620	No differences	Yes

[62]	4vs.2 + 1	Youth ^i^	20	HSR	2.3 ± 3.8	10.0 ± 6.7	335	Increase in larger pitch size	Yes

[62]	4vs.2 + 2	Youth ^i^	20	HSR	1.5 ± 6	7.8 ± 5.9	420	Increase in larger pitch size	Yes

[62]	4vs.2 + 3	Youth ^i^	20	HSR	3.5 ±	7.3 ± 4.3	109	Increase in larger pitch size	Yes

[93]	4vs.4	Youth ^a^	16	HSR	5.9 ± 6.0	30.9 ± 25.7	423.7	Increase in larger pitch size	Yes

[93]	4vs.4	Youth ^e^	18	HSR	3.9 ± 6.7	9.2 ± 10.4	135.9	Increase in larger pitch size	Yes

[93]	4vs.4	Youth ^i^	18	HSR	3.4 ± 4.3	5.7 ± 5.7	67.6	Increase in larger pitch size	Yes

[66]	4vs.4 + GK	Youth ^c^	36	TD	111.5 ± 10.9	128.7 ± 12.0	15.4	Increase in larger pitch size	Yes

[66]	4vs.4 + GK	Youth ^c^	36	HSR	4.6 ± 7.0	24.2 ± 20.4	426.1	Increase in larger pitch size	Yes

[66]	4vs.4 + GK	Youth ^e^	43	TD	121.3 ± 11.5	132.9 ± 13.8	9.6	Increase in larger pitch size	Yes

[66]	4vs.4 + GK	Youth ^e^	43	HSR	11.2 ± 11.7	43.8 ± 30.7	291.1	Increase in larger pitch size	Yes

[66]	4vs.4 + GK	Youth ^g^	28	TD	124.3 ± 9.6	134.4 ± 11.8	8.1	Increase in larger pitch size	Yes

[66]	4vs.4 + GK	Youth ^g^	28	HSR	12.8 ± 11.8	49.7 ± 28.6	288.3	Increase in larger pitch size	Yes

[66]	4vs.4 + GK	Youth ^i^	43	TD	128.3 ± 11.0	140.7 ± 12.0	9.7	Increase in larger pitch size	Yes

[66]	4vs.4 + GK	Youth ^i^	43	HSR	17.3 ± 14.4	50.3 ± 27.4	190.8	Increase in larger pitch size	Yes

[94]	4vs.4 + GK	Youth ^i^	10	TD	3444 ± 293	3517 ± 152	2.1	Increase in larger pitch size	Yes

[94]	4vs.4 + GK	Youth ^i^	10	HSR	31 ± 15	87 ± 51	180.6	Increase in larger pitch size	Yes

[96]	5vs.5	Youth ^a^	86	TD	1754 ± 237	1771 ± 314	1.0	Increase in larger pitch size	Yes
	8vs.8								

[96]	5vs.5	Youth ^a^	86	HSR	2 ± 6	6 ± 10	200.0	Increase in larger pitch size	Yes
	8vs.8								

[96]	8vs.8	Youth ^c^	86	TD	1821 ± 325	2038 ± 328	11.9	Increase in larger pitch size	Yes
	11vs.11								

[96]	8vs.8	Youth ^c^	86	HSR	2.5 ± NR	7.6 ± NR	204.0	Increase in larger pitch size	No Reason: no reported SD
	11vs.11								

SD: standard-deviation; TD: total distance; HSR: high speed running; ACC: accelerations; DEC: decelerations; %: percentage of difference; a: Under-11 group or below; b: Under-12; group; c: Under-13 group; d: Under-14 group; e; under-15 group; f: under-16 group; g: under-17 group; h: under-18 group; i: under-23 group; NR: non-reported

**FIG. 4:**
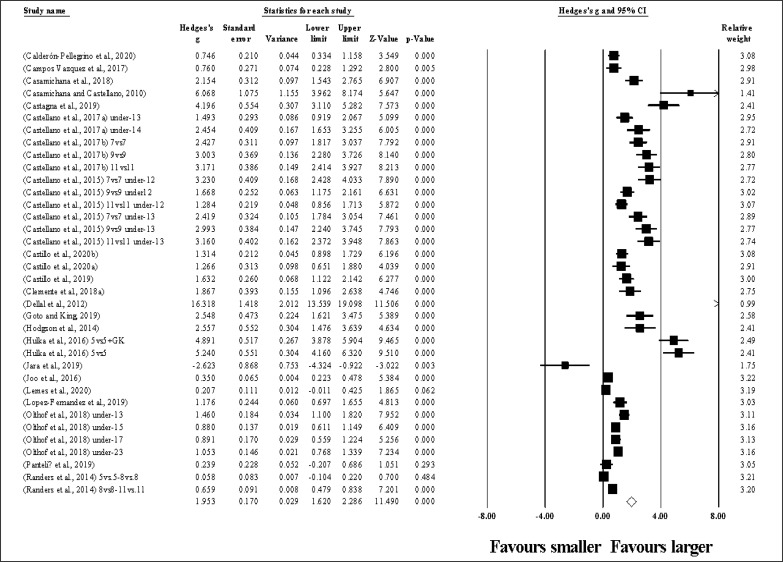
Forest plot of changes in total distance, in soccer players participating in small-sided games using smaller compared to larger pitch sizes. Values shown are effect sizes (Hedges’s g) with 95% confidence intervals (CI). The size of the plotted squares reflects the statistical relative weight of the study. The withe diamond reflects the overall result.

Thirty-six study groups provided data for TD, involving 36 smaller and 36 larger pitch sizes being compared (pooled *n* = 1.035). Results (Figure 4) showed that SSGs played at larger pitches induced greater TD compared to smaller pitches (ES = 1.95, large; 95% CI = 1.62 to 2.29; *p <* 0.001; *I*^2^ = 95.9%; Egger’s test *p <* 0.001, with a corrected value of ES = 2.49, 95% CI = 1.89 to 3.10; supplementary Figure 3).

**FIG. 5:**
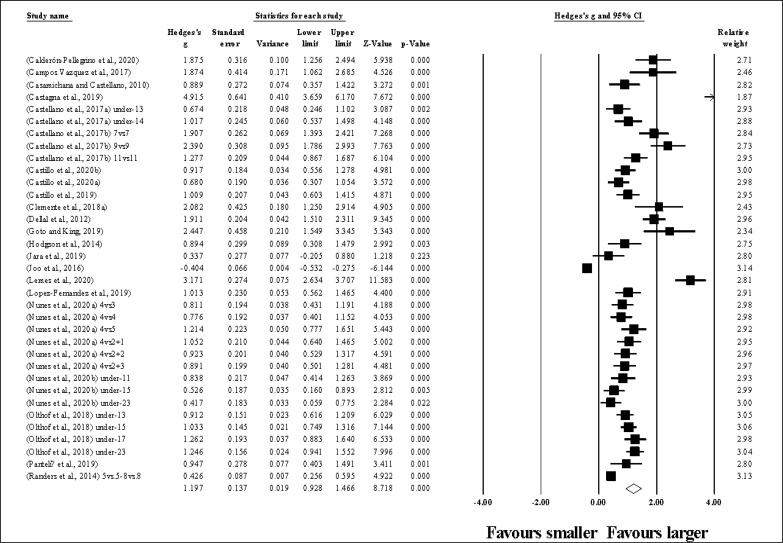
Forest plot of changes in high speed running distance, in soccer players participating in small-sided games using smaller compared to larger pitch sizes. Values shown are effect sizes (Hedges’s g) with 95% confidence intervals (CI). The size of the plotted squares reflects the statistical relative weight of the study. The withe diamond reflects the overall result.

Thirty-five study groups provided data for HSR, involving 35 smaller and 35 larger pitch sizes being compared (pooled *n* = 920). Results (Figure 5) showed that SSGs played at larger pitches induced greater HSR compared to smaller pitches (ES = 1.20, moderate; 95% CI = 0.93 to 1.47; *p <* 0.001; *I*^2^ = 94.4%; Egger’s test *p <* 0.001, with a corrected value of ES = 1.32, 95% CI = 0.95 to 1.70; supplementary Figure 4).

**FIG. 6:**
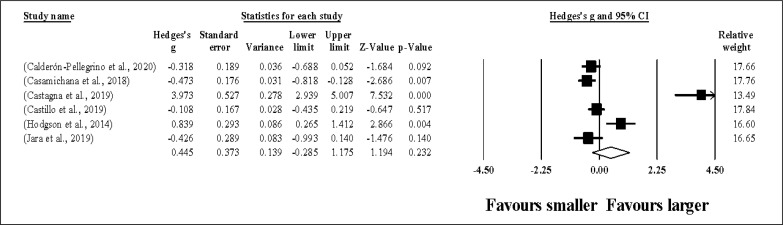
Forest plot of changes in acceleration actions, in soccer players participating in small-sided games using smaller compared to larger pitch sizes. Values shown are effect sizes (Hedges’s g) with 95% confidence intervals (CI). The size of the plotted squares reflects the statistical relative weight of the study. The withe diamond reflects the overall result.

Six study groups provided data for ACC, involving 6 smaller and 6 larger pitch sizes being compared (pooled *n* = 86). Results (Figure 6) showed that SSGs played at larger pitches induced similar ACC compared to smaller pitches (ES = 0.45, small; 95% CI = -0.29 to 1.18; *p* = 0.232; *I*^2^ = 93.5%; Egger’s test *p* = 0.040, with a corrected value of ES = 0.69, 95% CI = -0.17 to 1.55; supplementary Figure 5).

**FIG. 7:**
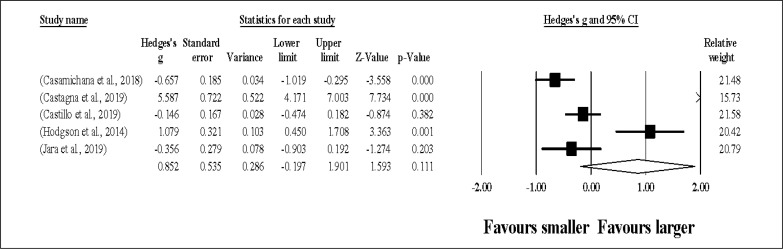
Forest plot of changes in deceleration actions, in soccer players participating in small-sided games using smaller compared to larger pitch sizes. Values shown are effect sizes (Hedges’s g) with 95% confidence intervals (CI). The size of the plotted squares reflects the statistical relative weight of the study. The withe diamond reflects the overall result.

Five study groups provided data for DEC, involving 5 smaller and 5 larger pitch sizes being compared (pooled *n* = 70). Results (Figure 7) showed that SSGs played at larger pitches induced similar DEC compared to smaller pitches (ES = 0.85, moderate; 95% CI = -0.20 to 1.90; *p* = 0.111; *I*^2^ = 95.3%; Egger’s test *p* = 0.049, with a corrected value of ES = 1.40, 95% CI = -0.17 to 2.97; supplementary Figure 6).

### Moderator analyses for TD, and HSR

The TD was greater at larger compared to smaller pitches when SSGs were played with either small (8 study groups; ES = 0.83; p < 0.001; *I*^2^ = 86.7%), medium (18 study groups; ES = 2.04; p < 0.001; I^2^ = 96.3%) and large (9 study groups; ES = 3.11; p < 0.001; *I*^2^ = 96.8%) formats, with significant sub-group difference between formats (*p <* 0.001).

The TD was greater at larger compared to smaller pitches when SSGs were played by young/youth (31 study groups; ES = 1.90; p < 0.001; *I*^2^ = 95.6%) and adult players (5 study groups; ES = 3.21; p = 0.007; *I*^2^ = 97.2%), without significant sub-group difference according to the age of the soccer players (*p* = 0.272).

The HSR was greater at larger compared to smaller pitches when SSGs were played with either small (17 study groups; ES = 1.09; p < 0.001; *I*^2^ = 83.4%), medium (13 study groups; ES = 0.93; p < 0.001; I^2^ = 94.9%) and large (4 study groups; ES = 1.87; p < 0.001; *I*^2^ = 71.8%) formats, with significant sub-group difference between formats (*p* = 0.009).

The HSR was greater at larger compared to smaller pitches when SSGs were played by young/youth (31 study groups; ES = 1.16; p < 0.001; *I*^2^ = 94.6%) and adult players (4 study groups; ES = 1.53; p = 0.001; *I*^2^ = 87.7%), without significant sub-group difference according to the age of the soccer players (*p* = 0.423).

Due to the limited number of study groups available for each moderator category, robust moderator analyses were precluded for ACC and DEC.

### Smaller0 vs. larger pitch sizes during SSG: effects on technical execution

A summary of the included studies and results of technical execution (passes and dribbles) reported in smaller and larger SSGs are provided in Table 7.

**TABLE 7:** Summary of the included studies and results of technical execution in smaller and larger pitch sizes.

Study	Format	Age category	N	Variable	Smaller Mean ± SD	Larger Mean ± SD	Larger-Smaller (%)*	Tendency of change	Included in the meta-analysis
[71]	5vs.5 + GK	Adults	10	Dribbles	13.9 ± 7.9	15.6 ± 6.8	12.2	Increase in larger pitch size	Yes

[71]	7vs.7 + GK	Adults	10	Dribbles	12.1 ± 6.5	11.3 ± 6.6	-6.6	Decrease in larger pitch size	Yes

[71]	5vs.5 + GK	Adults	10	Passes	32.8 ± 12.6	28.6 ± 9.0	-12.8	Decrease in larger pitch size	Yes

[71]	7vs.7 + GK	Adults	10	Passes	29.5 ± 9.6	26.9 ± 9.8	-8.8	Decrease in larger pitch size	Yes

[40]	5vs.5 + GK	Youth ^f^	10	Dribbles	5.2 ± 1.7	1.7 ± 0.8	-67.3	Decrease in larger pitch size	Yes

[40]	5vs.5 + GK	Youth ^f^	10	Passes	14.5 ± 6.6	18.7 ± 4.3	29.1	Increase in larger pitch size	No. Reason: results are the same as presented in the [40]

[18]	5vs.5 + GK	Youth ^f^	10	Dribbles	5.2 ± 1.7	1.7 ± 0.8	-67.3	Decrease in larger pitch size	No. Reason: results are the same as presented in the [40]

[18]	5vs.5 + GK	Youth ^f^	10	Passes	14.5 ± 6.6	18.7 ± 4.3	29.1	Increase in larger pitch size	No. Reason: results are the same as presented in the [40]

[84]	4vs.4	Adults	8	Dribbles	11.3 ± 8.5	12.8 ± 10.2	13.3	Increase in larger pitch size	Yes

[84]	4vs.4	Adults	8	Passes	74.6 ± 27.2	76.0 ± 35.3	1.9	Increase in larger pitch size	Yes

[61]	5vs.5 + GK	Youth ^i^	8	Dribbles	7.1 ± 2.6	6.9 ± 2.9	-2.8	Decrease in larger pitch size	Yes

[61]	5vs.5 + GK	Youth ^i^	8	Passes	23.1 ± 4.8	20.1 ± 3.0	-12.9	Decrease in larger pitch size	Yes

[87]	5vs.5 + GK	Youth ^g^	3	Passes	14 ± NR	8 ± NR	-42.9	Decrease in larger pitch size	No. Reason: no reported SD

[39]	8vs.8 + GK	Youth ^b^	149	Passes	13.8 ± 5.2	13.8 ± 6.4	0.0	No differences	Yes

[17]	5vs.5 + GK	Youth ^h^	8	Passes	71.5 ± 10.2	79.9 ± 13.5	11.7	Increase in larger pitch size	Yes

[17]	5vs.5 + GK	Youth ^h^	8	Dribbles	53.0 ± 10.1	62.2 ± 9.3	17.4	Increase in larger pitch size	Yes

[92]	5vs.5	Youth ^c^	10	Passes	18.6 ± 1.9	11.7 ± 1.6	-37.1	Decrease in larger pitch size	Yes

[92]	5vs.5	Youth ^c^	10	Dribbles	3.4 ± 0.8	3.6 ± 1.0	5.9	Increase in larger pitch size	Yes

[62]	4vs.3	Youth ^i^	20	Passes	9.3 ± 3.5	12.8 ± 5.4	37.6	Increase in larger pitch size	Yes

[62]	4vs.4	Youth ^i^	20	Passes	6.3 ± 3.2	10 ± 3.5	58.7	Increase in larger pitch size	Yes

[62]	4vs.5	Youth ^i^	20	Passes	7.3 ± 3.8	8.8 ± 4.6	20.5	Increase in larger pitch size	Yes

[62]	4vs.2 + 1	Youth ^i^	20	Passes	9.0 ± 4.0	9.3 ± 3.8	3.3	Increase in larger pitch size	Yes

[62]	4vs.2 + 2	Youth ^i^	20	Passes	8.0 ± 3.2	9.3 ± 3.8	16.3	Decrease in larger pitch size	Yes

[62]	4vs.2 + 3	Youth ^i^	20	Passes	9.0 ± 4.0	10.3 ± 3.2	14.4	Increase in larger pitch size	Yes

[93]	4vs.4	Youth ^a^	16	Passes	8.7 ± 4.9	11.3 ± 7.4	29.9	Increase in larger pitch size	Yes

[93]	4vs.4	Youth ^e^	18	Passes	7.1 ± 3.1	7.1 ± 2.7	0.0	No differences	Yes

[93]	4vs.4	Youth ^i^	18	Passes	8.0 ± 3.6	6.0 ± 4.1	-25.0	Decrease in larger pitch size	Yes

SD: standard-deviation; %: percentage of difference; a: Under-11 group; b: Under-12; group; c: Under-13 group; d: Under-14 group; e; under-15 group; f: under-16 group; g: under-17 group; h: under-18 group; i: under-23 group; NR: non-reported

**FIG. 8:**
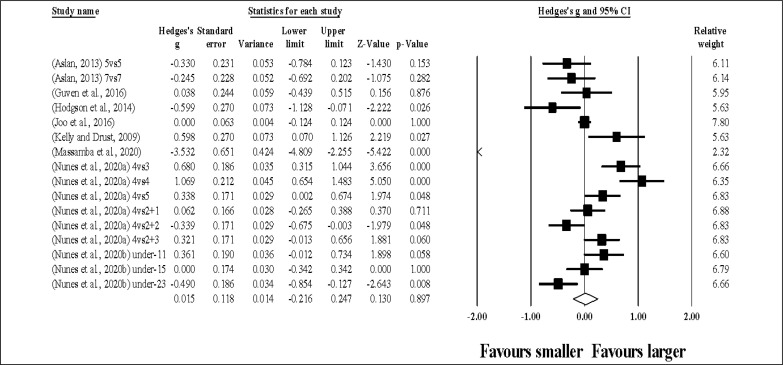
Forest plot of changes in passes, in soccer players participating in small-sided games using smaller compared to larger pitch sizes. Values shown are effect sizes (Hedges’s g) with 95% confidence intervals (CI). The size of the plotted squares reflects the statistical relative weight of the study. The withe diamond reflects the overall result.

**FIG. 9:**
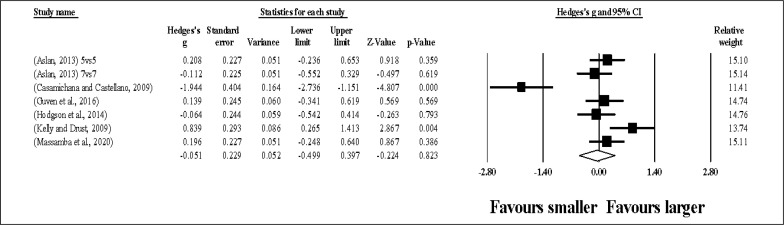
Forest plot of changes in dribbles, in soccer players participating in small-sided games using smaller compared to larger pitch sizes. Values shown are effect sizes (Hedges’s g) with 95% confidence intervals (CI). The size of the plotted squares reflects the statistical relative weight of the study. The withe diamond reflects the overall result.

Six-teen study groups provided data for passes, involving 16 smaller and 16 larger pitch sizes being compared (pooled *n* = 375). Results (Figure 8) showed that SSGs played at larger pitches induced similar passes compared to smaller pitches (ES = 0.02, trivial; 95% CI = -0.22 to 0.25; *p* = 0.897; *I*^2^ = 85.2%; Egger’s test *p* = 0.640, with a corrected value equal to the observed vaue; supplementary Figure 7). Seven study groups provided data for dribbling, involving 7 smaller and 7 larger pitch sizes being compared (pooled *n* = 64). Results (Figure 9) showed that SSGs played at larger pitches induced similar dribbles compared to smaller pitches (ES = -0.05, trivial; 95% CI = -0.50 to 0.40; *p* = 0.823; *I*^2^ = 82.0%; Egger’s test *p* = 0.159, with a corrected value of ES = -0.29, 95% CI = -0.76 to 0.18; supplementary Figure 8).

### Moderator analyses

The passes were similar at larger compared to smaller pitches when SSGs were played with either small (10 study groups; ES = 0.20; p = 0.153; *I*^2^ = 82.5%) and medium (6 study groups; ES = -0.42; p = 0.102; *I*^2^ = 87.9%) formats, with significant sub-group difference between formats (*p* = 0.034).

Due to the limited number of study groups available for each age category, robust moderator analyses were precluded for passes and dribbles, and the same for format of play in dribbles.

### Smaller vs. larger pitch sizes during SSG: effects on tactical behavior

A summary of the included studies and results of tactical behavior (centroid, stretch index and surface area) reported in smaller and larger SSGs are provided in Table 8.

**TABLE 8:** Summary of the included studies and results of tactical behavior in smaller and larger pitch sizes.

Study	Format	Age category	N	Variable	Smaller Mean ± SD	Larger Mean ± SD	Larger-Smaller (%)	Tendency of change	Included in the meta-analysis
[13]	7vs.7 + GK	Youth ^c^	14	Stretch index	8.6 ± 1.7	11.5 ± 2.3	33.7	Increase in larger pitch size	Yes

[13]	7vs.7 + GK	Youth ^d^	14	Stretch index	8.6 ± 2.5	10.1 ± 2.0	17.4	Increase in larger pitch size	Yes

[68]	11vs.11 + GK	Adults	10	Spatial exploration index	85.1 ± 1.6	68.6 ± 9.2	-19.4	Decrease in larger pitch size	No Reason: less than 3 studies reported the outcome

[81]	11vs.11 + GK	Adults	10	Stretch index	35.5 ± 9.5	45.8 ± 7.8	29.01	Increase in larger pitch size	Yes

[81]	11vs.11 + GK	Adults	10	Centroid (g)	24.5 ± 8.4	58.6 ± 9.6	139.2	Increase in larger pitch size	Yes

[81]	11vs.11 + GK	Adults	10	Centroid (l)	33.1 ± 6.1	36.8 ± 3.9	11.2	Increase in larger pitch size	Yes

[69]	4vs.4 + GK	Adults	10	Surface area	38 ± 31	34 ± 29	-10.5	Decrease in larger pitch size	Yes

[69]	4vs.4 + GK	Adults	10	Centroid (g)	1.5 ± 1.1	2.0 ± 1.2	33.3	Increase in larger pitch size	Yes

[69]	4vs.4 + GK	Adults	10	Centroid (l)	1.0 ± 0.8	1.1 ± 0.8	10.0	Increase in larger pitch size	Yes

[88]	5vs.5 + GK	Adults	3	Spatial exploration index	2.2 ± 0.3	3.2 ± 0.4	45.5	Increase in larger pitch size	No Reason: less than 3 studies reported the outcome

[88]	5vs.5 + GK	Adults	3	Predictive Ellipse Area	67.6 ± 18.8	129.1 ± 46.5	91.0	Increase in larger pitch size	No Reason: less than 3 studies reported the outcome

[88]	5vs.5 + GK	Adults	3	Standard Ellipse Area	11.3 ± 3.1	21.4 ± 7.8	89.4	Increase in larger pitch size	No Reason: less than 3 studies reported the outcome

[66]	4vs.4 + GK	Youth ^c^	36	Surface area	84.5 ± 8.7	143.2 ± 23.9	69.5	Increase in larger pitch size	Yes

[66]	4vs.4 + GK	Youth ^c^	36	Stretch index	4.6 ± 0.4	5.9 ± 1.8	28.3	Increase in larger pitch size	Yes

[66]	4vs.4 + GK	Youth ^c^	36	Width per length ratio	1.0 ± 0.2	1.1 ± 0.3	10.0	Increase in larger pitch size	No Reason: less than 3 studies reported the outcome

[66]	4vs.4 + GK	Youth ^e^	43	Surface area	94.8 ± 11.1	158.3 ± 34.2	67.0	Increase in larger pitch size	Yes

[66]	4vs.4 + GK	Youth ^e^	43	Stretch index	5.1 ± 0.4	6.2 ± 0.8	21.6	Increase in larger pitch size	Yes

[66]	4vs.4 + GK	Youth ^e^	43	Width per length ratio	0.9 ± 0.2	1.1 ± 0.3	22.2	Increase in larger pitch size	No Reason: less than 3 studies reported the outcome

[66]	4vs.4 + GK	Youth ^g^	28	Surface area	115.5 ± 30.9	146.4 ± 22.3	26.8	Increase in larger pitch size	Yes

[66]	4vs.4 + GK	Youth ^g^	28	Stretch index	5.3 ± 0.7	6.0 ± 0.6	13.2	Increase in larger pitch size	Yes

[66]	4vs.4 + GK	Youth ^g^	28	Width per length ratio	1.0 ± 0.1	1.1 ± 0.1	10.0	Increase in larger pitch size	No Reason: less than 3 studies reported the outcome

[66]	4vs.4 + GK	Youth ^i^	43	Surface area	101.2 ± 18.2	140.9 ± 27.0	39.2	Increase in larger pitch size	Yes

[66]	4vs.4 + GK	Youth ^i^	43	Stretch index	5.2 ± 0.6	5.9 ± 0.6	13.5	Increase in larger pitch size	Yes

[66]	4vs.4 + GK	Youth ^i^	43	Width per length ratio	0.9 ± 0.2	1.0 ± 0.2	11.1	Increase in larger pitch size	No Reason: less than 3 studies reported the outcome

[58]	4vs.4 + GK	Youth ^g^	20	Width per length ratio	1.1 ± 0.1	1.1 ± 0.2	0.0	No differences	No Reason: less than 3 studies reported the outcome

[58]	4vs.4 + GK	Youth ^g^	20	Surface area	119.5 ± 13.5	247.7 ± 44.6	107.3	Increase in larger pitch size	Yes

[58]	4vs.4 + GK	Youth ^g^	20	Centroid (g)	0.5 ± 0.1	0.6 ± 0.1	20.0	Increase in larger pitch size	Yes

[58]	4vs.4 + GK	Youth ^g^	20	Centroid (l)	0.4 ± 0.1	0.5 ± 0.1	25.0	Increase in larger pitch size	Yes

[58]	4vs.4 + GK	Youth ^f^	20	Width per length ratio	1.0 ± 0.5	1.5 ± 0.2	50.0	Increase in larger pitch size	No Reason: less than 3 studies reported the outcome

[58]	4vs.4 + GK	Youth ^f^	20	Surface area	121.2 ± 24.6	251.2 ± 46.3	107.3	Increase in larger pitch size	Yes

[58]	4vs.4 + GK	Youth ^f^	20	Centroid (g)	0.5 ± 0.1	0.5 ± 0.1	0.0	No differences	Yes

[58]	4vs.4 + GK	Youth ^f^	20	Centroid (l)	0.5 ± 0.1	0.4 ± 0.1	-20.0	Decrease in larger pitch size	Yes

[97]	6vs.6	Youth ^e^	24	Surface area	23.9 ± 7.0	28.8 ± 9.1	20.5	Increase in larger pitch size	Yes

SD: standard-deviation; g: goal-to-goal; l: lateral-to-lateral; %: percentage of difference; a: Under-11 group; b: Under-12; group; c: Under-13 group; d: Under-14 group; e; under-15 group; f: under-16 group; g: under-17 group; h: under-18 group; i: under-23 group

**FIG. 10:**
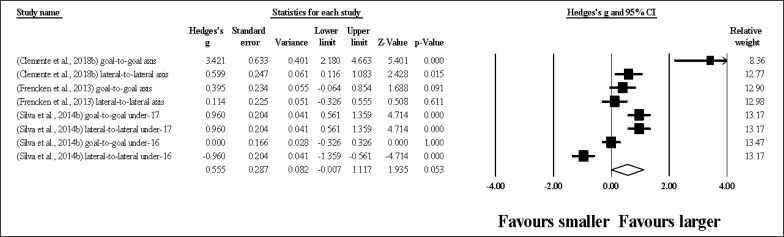
Forest plot of changes in centroid, in soccer players participating in small-sided games using smaller compared to larger pitch sizes. Values shown are effect sizes (Hedges’s g) with 95% confidence intervals (CI). The size of the plotted squares reflects the statistical relative weight of the study. The withe diamond reflects the overall result.

Eight study groups provided data for centroid, involving 8 smaller and 8 larger pitch sizes being compared (pooled *n* = 120). Results (Figure 10) showed that SSGs played at larger pitches induced similar centroid compared to smaller pitches (ES = 0.56, small; 95% CI = -0.01 to 1.12; *p* = 0.053; *I*^2^ = 92.2%; Egger’s test *p* = 0.151, with a corrected value of ES = 0.81, 95% CI = 0.11 to 1.51; supplementary Figure 9).

**FIG. 11:**
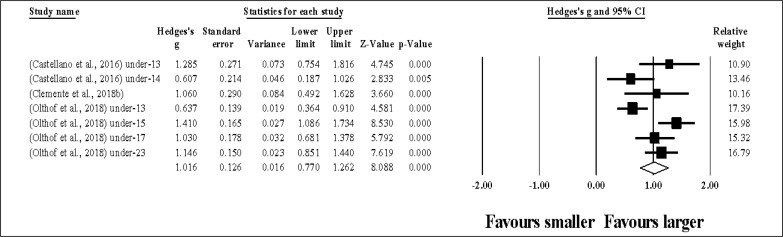
Forest plot of changes in stretch index, in soccer players participating in small-sided games using smaller compared to larger pitch sizes. Values shown are effect sizes (Hedges’s g) with 95% confidence intervals (CI). The size of the plotted squares reflects the statistical relative weight of the study. The withe diamond reflects the overall result.

Seven study groups provided data for stretch index, involving 7 smaller and 7 larger pitch sizes being compared (pooled *n* = 188). Results (Figure 11) showed that SSGs played at larger pitches induced greater stretch index compared to smaller pitches (ES = 1.02, moderate; 95% CI = 0.77 to 1.26; *p <* 0.001; *I*^2^ = 67.5%; Egger’s test *p* = 0.701, with a corrected value of ES = 0.89, 95% CI = 0.63 to 1.15; supplementary Figure 10).

**FIG. 12:**
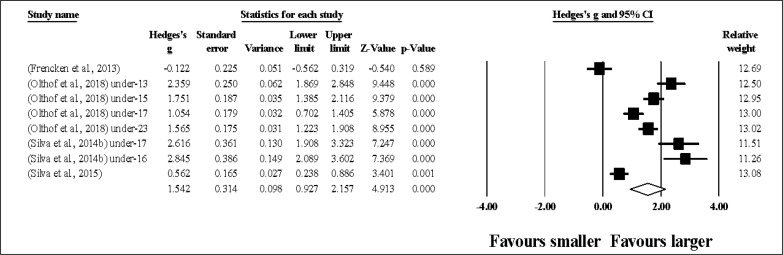
Forest plot of changes in surface area, in soccer players participating in small-sided games using smaller compared to larger pitch sizes. Values shown are effect sizes (Hedges’s g) with 95% confidence intervals (CI). The size of the plotted squares reflects the statistical relative weight of the study. The withe diamond reflects the overall result.

Eight study groups provided data for surface area, involving 8 smaller and 8 larger pitch sizes being compared (pooled *n* = 224). Results (Figure 12) showed that SSGs played at larger pitches induced greater surface area compared to smaller pitches (ES = 1.54, large; 95% CI = 0.93 to 2.16; *p <* 0.001; *I*^2^ = 94.0%; Egger’s test *p* = 0.164, with a corrected value of ES = 1.38, 95% CI = 0.78 to 1.97; supplementary Figure 11).

### Moderator analyses

Due to the limited number of study groups available for each moderator category, robust moderator analyses were precluded for centroid, stretch index and surface area.

## DISCUSSION

The current findings of this systematic review and meta-analysis revealed a meaningful effect of changing the pitch size in the physiological, physical, and tactical responses of soccer players during SSGs. Such an effect was not verified in technical responses.

### Smaller vs. larger pitch sizes during SSGs: Effects on physiological responses

The current systematic review and meta-analysis revealed that larger pitches meaningfully intensified exercise, reflected by the HR responses and RPE values of players. Additionally, a sub-group analysis revealed that this evidence was also significant in small, medium, and large formats of play, as well as in youth and adult players. Despite the high heterogeneity levels of meta-analysis and the experimental differences considering the pitch sizes, it was clear that larger pitch sizes generated greater HR (38 out of 42 study groups included) and RPE levels (33 out of 36 study groups included). Larger pitches were between 1.1x [39] and 3.7x [40] bigger than the smaller pitches, and all formats of play were covered.

It is reasonable to expect that greater physiological responses would occur in larger pitches since there more space to cover, albeit with less spatial exploration variability. However, larger pitches may make the game more structured [20] even though the space available allows each player to cover greater distances at a faster pace. This fact was confirmed in the current meta-analysis, as significantly greater distances were covered and greater dispersion between teammates was found on larger pitches. HR and RPE reflect external load demands [41], which could explain the meaningful physiological intensification occurring on larger pitches. Additionally, the fact that RPE is conditioned both by HR and external load demands (in particular, total distance) [42] further explains the association between larger pitches and higher HR and RPE scores.

Comparisons between larger and smaller pitch sizes were also executed while considering the format of play as moderators (for-mats of play were classified as small – 2 vs. 2 to 4 vs. 4; medium – 5 vs. 5 to 8 vs. 8; and large – 9 vs. 9 to 11 vs. 11). Interestingly, increasing the pitch size had similar effects in all formats (i.e., HR and RPE were significantly increased). Similarly, youth and adult players alike presented significantly greater values of HR and RPE on larger pitch sizes. Therefore, it can be argued that larger pitches foster more intense exercise than smaller pitches independent of the format of play or age group.

### Smaller vs. larger pitch sizes during SSGs: Effects on physical demands

The representative learning design and manipulation of task constraints are important pedagogical principles that coaches and practitioners should consider while planning training sessions [43]. Smaller and/or larger pitch sizes during SSGs can be utilized to achieve the main goal of the session. It is also known that the size of the pitch can be managed to simulate more or less the demands of the match. As an example, in a recent study [44], it was found that larger pitch sizes 156 to 182 m^2^ were the most similar to replicate match demands regarding the total distance, high-speed running, very high speed running, and sprinting.

For example, the results of our meta-analysis showed that SSGs played at larger pitches (range length: 30–108 m; range width: 20–68 m; range area per player: 37.5–334 m^2^) induced greater TD and HSR values than smaller configurations (range length: 10–68 m; range width: 13–68 m; range area per player: 15.6–199.75 m^2^), independent of the format of play and age. In addition, similar ACC and DEC values were observed during SSGs played on larger and smaller pitches.

A moderator analysis of the current study showed that high values of area per player (e.g., 300 m^2^) and reduced number of players (e.g., 7 vs. 7) increased the physical demands imposed on players during SSGs [14]. These findings should be considered by coaches during training planning, especially in youth academies and on training days before matches. A previous study demonstrated that simultaneously reducing absolute and relative area per player induced a higher frequency of offensive unity and increased the level of interaction between teammates [45]. Therefore, the increase in tactical performance and reduced physical demands (TD and HSR) during SSGs played with a low relative area per player suggest that this configuration can be beneficial in training sessions designed for youth and adult players of a low skill level, as the levels of task difficulty and complexity are easier to adjust [46]. On the other hand, according to the development of physical fitness and tactical skills, coaches and practitioners can increase the pitch size and formats. In addition, previous studies found that coaches periodized training contents to attain the highest weekly training load in the middle of the week (e.g., three days before a match) [47, 48]. Therefore, especially regarding starters (e.g., players who participate for at least 75 min in official matches), SSGs played on larger pitches are recommended in the middle of the week.

In youth and professional players, high-intensity activities (e.g., HSR and sprinting) are important physical variables in official matches [49–51]. In fact, using meta-analytical procedures, the current study provided robust conclusions about the advantages of larger SSGs in increasing TD and HSR compared to smaller SSGs. However, previous studies have provided a critical discussion about the specificity and representativeness of SSGs to stimulate the physical demands of official matches, which is worthy of further discussion [52]. Players tend to perform fewer high-intensity activities (e.g., > 19.8 km·h^-1^ or > 25.2 km·h^-1^) in SSGs than in official matches [53]. In contrast, accelerometry-based variables increase during SSGs [54] and achieve similar values to the peak periods of official matches [55]. Therefore, regarding physical demands, SSGs are not the same as official matches. External load monitoring and complementary exercise approaches (e.g., generic high-intensity running exercises) could be useful to ensure that distance- and accelerometry-based outcomes are achieved throughout the season [56]. Naturally, interactions of pitch sizes with other task constraints as rule modifications may produce different results for coaches. As an example, a recent study revealed that including mini-goals vs. using a ball possession match lead to lower values in physical demands [57].

### Smaller vs. larger pitch sizes during SSGs: Effects on technical execution

Analyzing the differences in the technical actions performed by players under different SSG rules may help coaches improve the propensity of the task to the main goal of the session. Knowledge on the impact of pitch size alterations on players’ technical execution is useful for better pedagogical planning. The results of the current systematic review and meta-analysis showed that the frequency of technical actions is not influenced by changing the pitch size.

Differences in tactical [58], physiological [59], and physical [60] responses were observed when the pitch size was changed. Also, smaller pitches seem to induce players to reduce the interpersonal distances between them. Together, these two factors could lead to differences in technical execution when the pitch size is manipulated, which contrasts the current results. The heterogeneity of the data and the type of variable measured are two main factors that might contribute to this effect.

Regarding the heterogeneity of the data, some studies indicated that the numbers of passes [61] and dribbles [40] are higher for SSGs played on smaller pitches, while others showed that these values are higher on larger pitches [17, 62]. We argue that other rules might have biased the results, leading to high heterogeneity. Specifically, the presence of a goalkeeper, the adoption of the offside rule, and the presence of floaters are examples of rules that were not homogeneously adopted across studies. For example, a previous study showed that numerically balanced SSGs required players to dribble more frequently than in formats with floaters [63]. Even though the smaller pitches brought players closer together (thus facilitating the execution of passes), the numerically balanced condition might have encouraged players to dribble instead of pass. Therefore, these contradictory effects, when expanded to other possible manipulations, might explain the high heterogeneity among results and the failure of some studies to detect an influence of changing the pitch size on technical execution.

Moreover, technical execution was primarily measured by accounting for the frequency of the events. Although this approach allows coaches to understand the propensity of each SSG to stimulate the main outcome of the session, it neglects the quality of the technical executions observed. Thus, coaches must also consider players’ skill levels [64] and task complexity [65] when deciding which formats to choose. Specifically, the same frequencies of technical actions could be observed even if differences in the quality of the execution are hidden. For example, since the players are closer to each other on smaller pitches [66], a higher percentage of successful passes could be expected, although dribbling could become more difficult because there is less available space. These results are not measurable by the current methodology, as most of the studies accounted only for the frequency and not the quality of technical actions. Therefore, adopting a contextual analysis of technical execution that includes performance indicators for each variable and accounts for skill efficacy and efficiency remains a challenge for future researchers in this topic.

### Smaller vs. larger pitch sizes during SSGs: Effects on tactical behavior

The selected studies on tactical behavior considered positional variables, collected by tracking techniques using devices such as GPS and LPM systems [58, 66]. All tactical variables were continuously measured during the SSGs and represent both with- and without-the-ball behaviors. These results revealed no differences in the centroid position when changing the pitch size. While, larger pitches induced higher values of the stretch index and surface area.

A previous study on this topic has shown that when a specific axis of the field (e.g., depth) is increased, the players tend to increase their exploration towards that axis [67], even if the relative area per player remains the same. This means that increases in the pitch size as a whole (not just on one axis) are expected to increase the spatial exploration along both axes—this explains the observed increase in the stretch index and the surface area in larger pitches. The larger the pitch, the further the players are expected to be from each other to cover larger distances to create scoring opportunities when attacking and prevent them when defending. On the other hand, no differences in the centroid position were observed when the pitch size was altered. In the current study, goal-to-goal and lateral-to-lateral axes were analyzed together due to the small sample, which might explain the absence of differences. Specifically, the goal-to-goal centroid difference seems to be more strongly affected by changing the pitch size than the lateral axis distance [58, 68, 69]. In the future, when more studies on each variable are available, a new investigation on this topic is recommended to test this hypothesis.

The positional differences resulting from changing the pitch size should be considered by coaches when designing training tasks using SSGs. Specifically, increasing the pitch size seems to increase the difficulty that players face when attempting to adequately occupy the most relevant spaces on the pitch. At this point, adjusting the tactical complexity to players’ current level is recommended [65]. For this reason, the increase in pitch size could be understood as a task constraint that should be progressively applied as the players get used to one specific format. In other words, when teaching young groups or introducing new tactical content to experienced groups, it could be beneficial to facilitate the tactical occupation by adopting smaller pitches—the pitch size can later be enlarged according to the development of players’ tactical skills on small pitches. Supporting this assumption, a previous study showed that enlarging the pitch size reduces the number of interactions that occur during SSGs [45]. This is a strong indicator of difficulty to adopt more complex offensive strategies when the pitch size is increased.

### Limitations, future research, and practical applications

Besides its contribution to the training process in soccer, the current review has limitations that must be considered. First, the high heterogeneity of the studies might be considered, as comparing studies methodologically different can increase the risk of bias. Therefore, a more in-depth investigation of SSGs is recommended to include studies with more similar experimental designs, thus reducing this bias. Also, none of the studies achieved a two-point score in the methodological quality assessment. This indicates that studies on SSGs should improve their methodology quality to adequately investigate the phenomenon. This issue is a challenge when conducting studies with high ecological validity, although recent advances in players’ monitoring allow better control of intervening variables when the SSGs are prescribed alongside regular training. Still, better descriptions of experimental protocols will increase the reproducibility of studies and, hence, improve the methodological quality of future research by allowing the replication of designs under different task conditions. It seems also important to emphasize the development of studies on the technical and tactical dimensions, as the small number of studies did not allow us to conduct a moderator analysis in the current systematic review. Finally, it seems important to consider the natural human variation occurring in SSGs which may play an important bias in case of no repeating measures in the experiments or in case of a high noise which may induce different results based on player’s participation [70].

In practical settings, SSGs with larger pitch sizes (e.g., > 250 m^2^ per player) can increase the physical demands imposed on players, especially TD and HSR. Considering that coaches periodize training contents in such a way that training load is increased until three days before the next match, larger pitches can be better during the middle of the week. In contrast, smaller pitches with optimal accelerometry loads can be a good option at the beginning of the week and/or until two days before the next match. In addition, SSGs with reduced load demands (e.g., smaller pitches associated with other task constraints) can facilitate the engagement of low-level young groups during training sessions.

Practical implications can be proposed concerning tactical and technical dimensions. Specifically, increasing the pitch size is not intended to impact the frequency of technical actions, although it significantly increases players’ area of occupation on the pitch. For this reason, smaller pitches should be preferably adopted in young groups, which will characterize a facilitated task condition and allow players to explore tactical solutions for emerging problems. On the other hand, larger pitches will create a challenging environment in which the spatial occupation will be more difficult, which can emphasize the development of collective tactical principles related to concentration and space creation.

## CONCLUSIONS

This systematic review revealed a clear effect of larger pitch sizes for increasing the intensification of internal load responses (HR and RPE), distances covered (total and HSR), and promoting the dispersion of players at a collective level (stretch index and surface area). These results were confirmed independently of the format of play and age group in terms of internal load and external load. Despite the heterogeneity of the pool of included articles, the individual results of each study provided clear support for these findings. On the other hand, meaningful differences were not evident between pitch sizes in terms of the numbers of accelerations, decelerations, passes, or dribbles performed. Based on the available evidence, larger pitch sizes can be recommended to increase the physiological and physical intensities of SSGs and promote collective dynamics occupying greater space to distance players.

## Conflicts of interest/Competing interests

The authors declare that they have no conflicts of interest relevant to the content of this review.

## References

[1] Stolen T, Chamari K, Castagna C, Wisloff U. Physiology of soccer: an update. Sport Med. 2005; 35(6):501–36. 10.2165/00007256-200535060-00004 15974635

[2] Paul DJ, Bradley PS, Nassis GP. Factors Affecting Match Running Performance of Elite Soccer Players: Shedding Some Light on the Complexity. Int J Sports Physiol Perform. 2015; 10(4):516–9. 25928752 10.1123/IJSPP.2015-0029

[3] Mendez-Villanueva A, Buchheit M. Physical capacity–match physical performance relationships in soccer: simply, more complex. Eur J Appl Physiol. 2011; 111(9):2387–9. 21331627 10.1007/s00421-011-1868-5

[4] Aquino R, Puggina EF, Alves IS, Garganta J. Skill-related performance in soccer: a systematic review. Hum Mov. 2017; 18(5):3–24.

[5] Costa IT da, Garganta J, Greco PJ, Mesquita I, Seabra A. Influence of Relative Age Effects and Quality of Tactical Behaviour in the Performance of Youth Soccer Players. Int J Perform Anal Sport. 2010; 10(2):82–97.

[6] Aquino R, Carling C, Maia J, Vieira LHP, Wilson RS, Smith N, Almeida R, Gonçalves LGC, Kalva-Filho CA, Garganta J, Puggina EF. Relationships between running demands in soccer match-play, anthropometric, and physical fitness characteristics: a systematic review. Int J Perform Anal Sport. 2020; 20(3):534–555.

[7] Clemente FM, Sarmento H. The effects of small-sided soccer games on technical actions and skills: A systematic review. Hum Mov. 2020; 21(3):100–19.

[8] Clemente FM, Afonso J, Castillo D, Arcos AL, Silva AF, Sarmento H. The effects of small-sided soccer games on tactical behavior and collective dynamics: A systematic review. Chaos, Solitons & Fractals. 2020; 134:109710.

[9] Sarmento H, Clemente FM, Harper LD, Costa IT da, Owen A, Figueiredo AJ. Small sided games in soccer – a systematic review. Int J Perform Anal Sport. 2018; 18(5):693–749.

[10] Davids K, Araújo D, Correia V, Vilar L, Araú Jo D, Correia V, Vilar L. How small-sided and conditioned games enhance acquisition of movement and decision-making skills. Exerc Sport Sci Rev. 2013; 41(3):154–61. 23558693 10.1097/JES.0b013e318292f3ec

[11] Clemente FM, Afonso J, Sarmento H. Small-sided games: An umbrella review of systematic reviews and meta-analyses. PLoS One. 2021; 16(2): e0247067. 33577611 10.1371/journal.pone.0247067 PMC7880470

[12] Hill-Haas S V, Dawson BT, Impellizzeri FM, Coutts AJ. Physiology of small-sided games training in football: A systematic review. Sport Med. 2011; 41(3):199–220. 10.2165/11539740-000000000-00000 21395363

[13] Castellano J, Puente A, Echeazarra I, Usabiaga O, Casamichana D. Number of players and relative pitch area per player: comparing their influence on heart rate and physical demands in under-12 and under-13 football players. PLoS One. 2016; 11(1):e0127505. 26752422 10.1371/journal.pone.0127505 PMC4709045

[14] Castellano J, Puente A, Echeazarra I, Casamichana D. Influence of the number of players and the relative pitch area per player on heart rate and physical demands in youth soccer. J Strength Cond Res. 2015; 29(6):1683–91. 25474336 10.1519/JSC.0000000000000788

[15] Martone D, Giacobbe M, Capobianco A, Imperlini E, Mancini A, Capasso M, Buono P, Orrù S. Exercise Intensity and Technical Demands of Small-Sided Soccer Games for Under-12 and Under-14 Players. J Strength Cond Res. 2017; 31(6):1486–92. 28538296 10.1519/JSC.0000000000001615

[16] Silva P, Vilar L, Davids K, Araújo D, Garganta J. Sports teams as complex adaptive systems: manipulating player numbers shapes behaviours during football small-sided games. Springerplus. 2016; 5(1):191. 10.1186/s40064-016-1813-5 PMC4769238 27026887

[17] Kelly DM, Drust B. The effect of pitch dimensions on heart rate responses and technical demands of small-sided soccer games in elite players. J Sci Med Sport. 2009; 12(4):475–9. 18356102 10.1016/j.jsams.2008.01.010

[18] Casamichana D, Castellano J. Time– motion, heart rate, perceptual and motor behaviour demands in small-sides soccer games: Effects of pitch size. J Sports Sci. 2010; 28(14):1615–23. 21077005 10.1080/02640414.2010.521168

[19] Lemes JCJC, Luchesi M, Diniz LBFLBF, Bredt SDGTSDGT, Chagas MHMH, Praça GMGM. Influence of pitch size and age category on the physical and physiological responses of young football players during small-sided games using GPS devices. Res Sport Med. 2020; 28(2):206–16. 10.1080/15438627.2019.1643349 31303051

[20] Silva P, Aguiar P, Duarte R, Davids K, Araújo D, Garganta J. Effects of Pitch Size and Skill Level on Tactical Behaviours of Association Football Players During Small-Sided and Conditioned Games. Int J Sport Sci Coach. 2014; 9(5):993–1006. 10.1080/02640414.2014.961950 25356995

[21] Bujalance-Moreno P, Latorre-Román PÁ, García-Pinillos F. A systematic review on small-sided games in football players: Acute and chronic adaptations. J Sports Sci. 2019; 37(8):921–49. 30373471 10.1080/02640414.2018.1535821

[22] Francesco Sgrò, Salvatore Bracco, Salvatore Pignato, Mario Lipoma. Small-Sided Games and Technical Skills in Soccer Training: Systematic Review and Implications for Sport and Physical Education Practitioners. J Sport Sci. 2018; 6(1):9–19.

[23] Green S, Higgins J. Cochrane handbook for systematic reviews of interventions. NJ, USA: John Wiley & Sons: Hoboken; 2005.

[24] Moher D, Liberati A, Tetzlaff J, Altman DG. Preferred Reporting Items for Systematic Reviews and Meta-Analyses: The PRISMA Statement. PLoS Med. 2009; 6(7):e1000097. 19621072 10.1371/journal.pmed.1000097 PMC2707599

[25] Rico-González M, Pino-Ortega J, Clemente F, Los Arcos A. Guidelines for performing systematic reviews in sports science. Biol Sport. 2022; 39(2):463–71. 35309539 10.5114/biolsport.2022.106386 PMC8919872

[26] Collaboration C. Data Extraction Template for Included Studies. 2016 [cited 2021 Jan 2]. Available from: https://cccrg.cochrane.org/sites/cccrg.cochrane.org/files/public/uploads/det_2015_revised_final_june_20_2016_nov_29_revised.doc

[27] Slim K, Nini E, Forestier D, Kwiatkowski F, Panis Y, Chipponi J. Methodological index for non-randomized studies (MINORS): development and validation of a new instrument. ANZ J Surg. 2003; 73(9):712–6. 12956787 10.1046/j.1445-2197.2003.02748.x

[28] Valentine JC, Pigott TD, Rothstein HR. How Many Studies Do You Need? J Educ Behav Stat. 2010; 35(2):215–47.

[29] Abt G, Boreham C, Davison G, Jackson R, Nevill A, Wallace E, Williams M. Power, precision, and sample size estimation in sport and exercise science research. J Sports Sci. 2020; 38(17):1933–5. 32558628 10.1080/02640414.2020.1776002

[30] Zouhal H, Hammami A, Tijani JM, Jayavel A, de Sousa M, Krustrup P, Sghaeir Z, Granacher U, Ben Abderrahman A. Effects of Small-Sided Soccer Games on Physical Fitness, Physiological Responses, and Health Indices in Untrained Individuals and Clinical Populations: A Systematic Review. Sport Med. 2020; 50(5):987–1007. 10.1007/s40279-019-01256-w 31989457

[31] Deeks JJ, Higgins JP, Altman DG. Analysing data and undertaking meta-analyses. In: Higgins JP, Green S, editors. Cochrane Handbook for Systematic Reviews of Interventions. The Cochrane Collaboration; 2008. p. 243–96.

[32] Kontopantelis E, Springate DA, Reeves D. A Re-Analysis of the Cochrane Library Data: The Dangers of Unobserved Heterogeneity in Meta-Analyses. Friede T, editor. PLoS One. 2013; 8(7):e69930. 23922860 10.1371/journal.pone.0069930 PMC3724681

[33] Hopkins WG, Marshall SW, Batterham AM, Hanin J. Progressive Statistics for Studies in Sports Medicine and Exercise Science. Med Sci Sport Exerc [Internet]. 2009; 41(1):3–13. 10.1249/MSS.0b013e31818cb278 19092709

[34] Higgins JPT, Thompson SG. Quantifying heterogeneity in a meta-analysis. Stat Med. 2002; 21(11):1539–58. 12111919 10.1002/sim.1186

[35] Egger M, Smith GD, Schneider M, Minder C. Bias in meta-analysis detected by a simple, graphical test. BMJ. 1997 Sep 13; 315(7109):629–34. 9310563 10.1136/bmj.315.7109.629 PMC2127453

[36] Duval S, Tweedie R. Trim and Fill: A Simple Funnel-Plot-Based Method of Testing and Adjusting for Publication Bias in Meta-Analysis. Biometrics. 2000; 56(2):455–63. 10877304 10.1111/j.0006-341x.2000.00455.x

[37] Shi L, Lin L. The trim-and-fill method for publication bias. Medicine (Baltimore) [Internet]. 2019; 98(23):e15987. 31169736 10.1097/MD.0000000000015987 PMC6571372

[38] Owen AL, Wong DP, Paul D, Dellal A. Physical and Technical Comparisons between Various-Sided Games within Professional Soccer. Int J Sports Med. 2014; 35(4):286–92. 24022576 10.1055/s-0033-1351333

[39] Joo CH, Hwang-Bo K, Jee H. Technical and Physical Activities of Small-Sided Games in Young Korean Soccer Players. J Strength Cond Res. 2016; 30(8):2164–73. 26808851 10.1519/JSC.0000000000001319

[40] Casamichana DC, Castellano J. Análisis de los diferentes espacios individuales de interacción y los efectos en las conductas motrices de los jugadores: aplicaciones al entrenamiento en fútbol. Eur J Hum Mov. 2009; 23:143–67.

[41] McLaren SJ, Macpherson TW, Coutts AJ, Hurst C, Spears IR, Weston M. The Relationships Between Internal and External Measures of Training Load and Intensity in Team Sports: A Meta-Analysis. Sport Med. 2018; 48(3):641–58. 10.1007/s40279-017-0830-z 29288436

[42] Casamichana D, Castellano J, Calleja-Gonzalez J, San Román J, Castagna C. Relationship Between Indicators of Training Load in Soccer Players. J Strength Cond Res. 2013; 27(2):369–74. 22465992 10.1519/JSC.0b013e3182548af1

[43] Chow JY. Nonlinear Learning Underpinning Pedagogy: Evidence, Challenges, and Implications. Quest. 2013; 65(4):469–84.

[44] Riboli A, B.H. Olthof S, Esposito F, Coratella G. Training elite youth soccer players: area per player in small-sided games to replicate the match demands. Biol Sport. 2022; 39(3):579–98. 35959338 10.5114/biolsport.2022.106388 PMC9331353

[45] Moreira PED, Barbosa GF, Murta CDCF, Morales JCP, Bredt SDGT, Praça GM, Greco PJ. Network analysis and tactical behaviour in soccer small-sided and conditioned games: influence of absolute and relative playing areas on different age categories. Int J Perform Anal Sport. 2019; 20(1):1–14.

[46] Machado JC, Barreira D, Teoldo I, Travassos B, Júnior JB, Santos JJOL Dos, Scaglia AJ, Junior JB, Santos JJOL Dos, Scaglia AJ. How Does the Adjustment of Training Task Difficulty Level Influence Tactical Behavior in Soccer? Res Q Exerc Sport. 2019; 90(3):403–16. 31157599 10.1080/02701367.2019.1612511

[47] Anderson L, Orme P, Di Michele R, Close GGL, Milson J, Morgans R, Morton JP, Michele R Di, Close GGL, Milsom J, Morgans R, Drust B, Morton JP. Quantification of Seasonal-Long Physical Load in Soccer Players With Different Starting Status From the English Premier League: Implications for Maintaining Squad Physical Fitness. Int J Sports Physiol Perform. 2016; 11(8):1038–46. 26915393 10.1123/ijspp.2015-0672

[48] Los Arcos A, Mendez-Villanueva A, Martínez-Santos R. In-season training periodization of professional soccer players. Biol Sport. 2017; 2:149–55. 10.5114/biolsport.2017.64588 PMC5424454 28566808

[49] Carling C, Bloomfield J, Nelsen L, Reilly T. The role of motion analysis in elite soccer: contemporary performance measurement techniques and work rate data. Sports Med. 2008; 38(10):839–62. 18803436 10.2165/00007256-200838100-00004

[50] Palucci Vieira LH, Carling C, Barbieri FA, Aquino R, Santiago PRP. Match Running Performance in Young Soccer Players: A Systematic Review. Sport Med. 2019; 49(2):289–318. 10.1007/s40279-018-01048-8 30671900

[51] Varley MC, Gregson W, McMillan K, Bonanno D, Stafford K, Modonutti M, Di Salvo V. Physical and technical performance of elite youth soccer players during international tournaments: influence of playing position and team success and opponent quality. Sci Med Footb. 2017; 1(1):18–29.

[52] Clemente FM. The Threats of Small-Sided Soccer Games: A Discussion About Their Differences With the Match External Load Demands and Their Variability Levels. Streng Cond J. 2020; 42(3):100–105.

[53] Casamichana D, Castellano J, Castagna C. Comparing the Physical Demands of Friendly Matches and Small-Sided Games in Semiprofessional Soccer Players. J Strength Cond Res. 2012; 26(3):837–843. 22310516 10.1519/JSC.0b013e31822a61cf

[54] Clemente FM, Sarmento H, Rabbani A, Van Der Linden CMI (Niels), Kargarfard M, Costa IT. Variations of external load variables between medium- and large-sided soccer games in professional players. Res Sport Med. 2019; 27(1):50–9. 10.1080/15438627.2018.1511560 30129780

[55] Dalen T, Sandmæl S, Stevens TG., Hjelde GH, Kjøsnes TN, Wisløff U. Differences in Acceleration and High-Intensity Activities Between Small-Sided Games and Peak Periods of Official Matches in Elite Soccer Players. J Strength Cond Res. 2019; 35(7):2018–2024. 10.1519/JSC.0000000000003081 30741867

[56] Clemente FM, Sarmento H. Combining small-sided soccer games and running-based methods: A systematic review. Biol Sport. 2021; 38(1):617–27. 34937972 10.5114/biolsport.2021.102932 PMC8670792

[57] Bujalance-Moreno P, Latorre-Román PÁ, Martínez-Amat A, García-Pinillos F. Small-sided games in amateur players: rule modification with mini-goals to induce lower external load responses. Biol Sport. 2022; 39(2):367–77. 35309544 10.5114/biolsport.2022.105336 PMC8919881

[58] Silva P, Duarte R, Sampaio J, Aguiar P, Davids K, Araujo D, Garganta J, Araújo D, Garganta J. Field dimension and skill level constrain team tactical behaviours in small-sided and conditioned games in football. J Sports Sci. 2014; 32(20):1888–96. 25356995 10.1080/02640414.2014.961950

[59] Halouani J, Chtourou H, Dellal A, Chaouachi A, Chamari K. The effects of game types on intensity of small-sided games among pre-adolescent youth football players. Biol Sport. 2017; 34(2):157–62. 28566809 10.5114/biolsport.2017.64589 PMC5424455

[60] Lemes JC, Luchesi M, Diniz LBF, Bredt SDGT, Chagas MH, Praça GM. Influence of pitch size and age category on the physical and physiological responses of young football players during small-sided games using GPS devices. Res Sport Med. 2020; 28(2):206–216. 10.1080/15438627.2019.1643349 31303051

[61] Hodgson C, Akenhead R, Thomas K. Time-motion analysis of acceleration demands of 4v4 small-sided soccer games played on different pitch sizes. Hum Mov Sci. 2014; 33(1):25–32. 24576705 10.1016/j.humov.2013.12.002

[62] Nunes NA, Gonçalves B, Coutinho D, Nakamura FY, Travassos B. How playing area dimension and number of players constrain football performance during unbalanced ball possession games. Int J Sport Sci Coach. 2020; 16(2):334–343.

[63] Sanchez-Sanchez J, Hernández D, Casamichana D, Martínez-Salazar C, Ramirez-Campillo R, Sampaio J. Heart Rate, Technical Performance, and Session-RPE in Elite Youth Soccer Small-Sided Games Played With Wildcard Players. J Strength Cond Res. 2017; 31(10):2678–85. 27930455 10.1519/JSC.0000000000001736

[64] Silva P, Duarte R, Sampaio J, Aguiar P, Davids K, Araujo D, Garganta J. Field dimension and skill level constrain team tactical behaviours in small-sided and conditioned games in football. J Sports Sci. 2014; 32(20):1888–96. 25356995 10.1080/02640414.2014.961950

[65] Machado JC, Barreira D, Teoldo I, Serra-Olivares J, Góes A, José Scaglia A. Tactical Behaviour of Youth Soccer Players: Differences Depending on Task Constraint Modification, Age and Skill Level. J Hum Kinet. 2020; 75(1):225–38. 33312309 10.2478/hukin-2020-0051 PMC7706672

[66] Olthof SBH, Frencken WGP, Lemmink KAPM. Match-derived relative pitch area changes the physical and team tactical performance of elite soccer players in small-sided soccer games. J Sports Sci. 2018; 36(14):1557–63. 29125029 10.1080/02640414.2017.1403412

[67] Gollin M, Alfero S, Daga AF. Manipulation of playing field’s length/width ratio and positional players’ orientation: activity profile and motor behavior demands during positional possession small sided games in young élite soccer players. Int J Sport Sci. 2016; 6(3):106–15.

[68] Clemente FM, Owen A, Serra-Olivares J, Correia A, Bernardo Sequeiros J, Silva FGM, Martins FML. The effects of large-sided soccer training games and pitch size manipulation on time–motion profile, spatial exploration and surface area: Tactical opportunities. Proc Inst Mech Eng Part P J Sport Eng Technol. 2018; 232(2):160–5.

[69] Frencken W, Van Der Plaats J, Visscher C, Lemmink K. Size matters: Pitch dimensions constrain interactive team behaviour in soccer. J Syst Sci Complex. 2013; 26(1):85–93.

[70] Younesi S, Rabbani A, Clemente F, Sarmento H, J. Figueiredo A. Session-to-session variations in external load measures during small-sided games in professional soccer players. Biol Sport. 2021; 38(2):185–93. 34079163 10.5114/biolsport.2020.98449 PMC8139343

[71] Aslan A. Cardiovascular Responses, Perceived Exertion and Technical Actions During Small-Sided Recreational Soccer: Effects of Pitch Size and Number of Players. J Hum Kinet. 2013; 38:95–105. 24233236 10.2478/hukin-2013-0049 PMC3827765

[72] Calderón-Pellegrino G, Paredes-Hernández V, Sánchez-Sánchez J, García-Unanue J, Gallardo L. Effect of the fatigue on the physical performance in different small-sided games in football players. J Strength Cond Res. 2020; 34(8):2338–46. 30299393 10.1519/JSC.0000000000002858

[73] Campos Vazquez MA, Casamichana Gomez D, Suarez Arrones L, Gonzalez Jurado JA, Toscano Bendala FJ, Leon Prados JA. Medium-sided games in soccer: physical and heart rate demands throughout successive working periods. J Hum Sport Exerc. 2017; 12(1):129–41.

[74] Casamichana D, Bradley PS, Castellano J. Influence of the Varied Pitch Shape on Soccer Players Physiological Responses and Time-Motion Characteristics During Small-Sided Games. J Hum Kinet. 2018; 64(1):171–80. 30429909 10.1515/hukin-2017-0192 PMC6231353

[75] Castagna C, D’Ottavio S, Cappelli S, Araujo Povoas SC. The Effects of Long Sprint Ability-Oriented Small-Sided Games Using Different Ratios of Players to Pitch Area on Internal and External Load in Soccer Players. Int J Sports Physiol Perform. 2019; 14(9):1265–72. 10.1123/ijspp.2018-0645 30860405

[76] Castellano J, Echeazarra I, Estefano I. Comparison of the physical demands in sub13 and sub14 football players on a 7-a-side game played with different lengths. Cult Cienc Y Deport. 2017; 12(34):55–65.

[77] Castellano J, Fernández E, Echeazarra I, Barreira D, Garganta J. Influencia de la longitud del campo en los comportamientos ínter e intra-equipo en jóvenes jugadores de fútbol. Ann Psychol. 2017; 33(3):486–96.

[78] Castillo D, Raya-González J, Manuel Clemente F, Yanci J. The influence of youth soccer players’ sprint performance on the different sided games’ external load using GPS devices. Res Sport Med. 2020; 28(2):194–205. 10.1080/15438627.2019.1643726 31307236

[79] Castillo D, Raya-González J, Manuel Clemente F, Yanci J. The influence of offside rule and pitch sizes on the youth soccer players’ small-sided games external loads. Res Sport Med. 2020; 28(3):324–38. 10.1080/15438627.2020.1739687 32183556

[80] Castillo D, Rodriguez-Fernandez A, Nakamura FY, Sanchez-Sanchez J, Ramirez-Campillo R, Yanci J, Zubillaga A, Raya-González J. Influence of different small-sided game formats on physical and physiological demands and physical performance in young soccer players. J Strength Cond Res. 2021;35(8):2287-93. 30908372 10.1519/JSC.0000000000003114

[81] Clemente FM, Sequeiros JB, Correia A, Serra-Olivares J, Gonzalez-Villora S, Silva F, Lourenco Martins FM. How dots behave in two different pitch sizes? Analysis of tactical behavior based on position data in two soccer field sizes. Ricyde-Revista Int Ciencias Del Deport. 2018; 14(51):16–28.

[82] Dellal A, Owen A, Wong DPP, Krustrup P, van Exsel M, Mallo J. Technical and physical demands of small vs. large sided games in relation to playing position in elite soccer. Hum Mov Sci. 2012; 31(4):957–69. 22341858 10.1016/j.humov.2011.08.013

[83] Goto H, King JA. High-Intensity Demands of 6-a-Side Small-Sided Games and 11-a-Side Matches in Youth Soccer Players. Pediatr Exerc Sci. 2019; 31(1):85–90. 30500316 10.1123/pes.2018-0122

[84] Guven F, Erkmen N, Aktas S, Taskin C. Small-sided games in football: effect of field sizes on technical parameters. Sport Sci Pract Asp. 2016; 13(2):35–43.

[85] Halouani J, Chtourou H, Dellal A, Chaouachi A, Chamari K. The effects of game types on intensity of small-sided games among pre-adolescent youth football players. Biol Sport. 2017; 34(2):157–62. 28566809 10.5114/biolsport.2017.64589 PMC5424455

[86] Hulka K, Weisser R, Belka J. Effect of the Pitch Size And Presence of Goalkeepers on the Work Load of Players During Small-Sided Soccer Games. J Hum Kinet. 2016; 51(1):175–81. 28149380 10.1515/hukin-2015-0180 PMC5260560

[87] Jara D, Ortega E, Gómez MA, Baranda PSD. Effect of Pitch Size on Technical-Tactical Actions of the Goalkeeper in Small-Sided Games. J Hum Kinet. 2018; 62(1):157–66. 29922387 10.1515/hukin-2017-0167 PMC6006530

[88] Jara D, Ortega E, Gomez-Ruano M-A, Weigelt M, Nikolic B, Sainz de Baranda P. Physical and Tactical Demands of the Goalkeeper in Football in Different Small-Sided Games. Sensors. 2019; 19(16). 10.3390/s19163605 PMC6719184 31430940

[89] Köklü Y, Albayrak M, Keysan H, Alemdaroğlu U, Dellal A. Improvement of the physical conditioning of young soccer players by playing small-sided games on different pitch size – special reference to physiological responses. Kinesiology. 2013; 45(1):41–7.

[90] Lopez-Fernandez J, Gallardo L, Fernandez-Luna A, Villacanas V, Garcia-Unanue J, Sanchez-Sanchez J. Pitch Size and Game Surface in Different Small-Sided Games. Global Indicators, Activity Profile, and Acceleration of Female Soccer Players. J strength Cond Res. 2019; 33(3):831–8. 28658077 10.1519/JSC.0000000000002090

[91] Lopez-Fernandez J, Sanchez-Sanchez J, Rodriguez-Canamero S, Ubago-Guisado E, Colino E, Gallardo L. Physiological responses, fatigue and perception of female soccer players in small-sided games with different pitch size and sport surfaces. Biol Sport. 2018; 35(3):291–9. 30449947 10.5114/biolsport.2018.77829 PMC6224843

[92] Massamba A, Dufour SP, Favret F, Hureau TJ. Small-Sided Games Are Not as Effective as Intermittent Running to Stimulate Aerobic Metabolism in Prepubertal Soccer Players. Int J Sports Physiol Perform. 2020; 1–7. 10.1123/ijspp.2019-0966 32820134

[93] Nunes NA, Gonçalves B, Davids K, Esteves P, Travassos B. How manipulation of playing area dimensions in ball possession games constrains physical effort and technical actions in under-11, under-15 and under-23 soccer players. Res Sport Med. 2020; 16(2):334–343. 10.1080/15438627.2020.1770760 32452730

[94] Pantelić S, Rađa A, Erceg M, Milanović Z, Trajković N, Stojanović E, Krustrup P, Randers MB. Relative pitch area plays an important role in movement pattern and intensity in recreational male football. Biol Sport. 2019; 36(2):119–24. 31223188 10.5114/biolsport.2019.81113 PMC6561223

[95] Rampinini E, Impellizzeri FM, Castagna C, Abt G, Chamari K, Sassi A, Marcora SM. Factors influencing physiological responses to small-sided soccer games. J Sports Sci. 2007; 25(6):659–66. 17454533 10.1080/02640410600811858

[96] Randers MB, Andersen TB, Rasmussen LS, Larsen MN, Krustrup P. Effect of game format on heart rate, activity profile, and player involvement in elite and recreational youth players. Scand J Med Sci Sports. 2014; 24:17–26. 24944130 10.1111/sms.12255

[97] Silva P, Esteves P, Correia V, Davids K, Araujo D, Garganta J. Effects of manipulations of player numbers vs. field dimensions on inter-individual coordination during small-sided games in youth football. Int J Perform Anal Sport. 2015; 15(2):641–59.

[98] Vilar L, Duarte R, Silva P, Chow JY, Davids K. The influence of pitch dimensions on performance during small-sided and conditioned soccer games. J Sports Sci. 2014; 32(19):1751–9. 24915106 10.1080/02640414.2014.918640

